# State‐of‐Art in Studying the Public Health Effects of Heat: A Literature Review

**DOI:** 10.1002/gch2.202500381

**Published:** 2025-09-24

**Authors:** Lorenzo Gianquintieri, Enrico Gianluca Caiani

**Affiliations:** ^1^ Politecnico di Milano Electronics, Information and Bioengineering Department Milano 20133 Italy; ^2^ Istituto Auxologico Italiano IRCCS S. Luca Hospital Milano 20149 Italy

**Keywords:** adaptation, climate change, DLNM, heat, heatwave, literature review, risk assessment

## Abstract

The impact of heat and heatwaves on human health constitutes a significant hazard, disproportionately affecting various regions worldwide and continuously exacerbating due to climate change. While human pathophysiology is well understood, addressing this challenge from a public‐health perspective is essential to generate population‐level insights and provide policymakers with evidence‐based guidance. This calls for an interdisciplinary “environmental epidemiology” approach, which embraces multiple areas including (but not limited to) health sciences, geomatics, data‐science, environmental analyses, and social sciences. Since the early 2000s, scientific production on this topic is increasing exponentially. This review aims to provide a narrative synthesis of the most relevant public‐health studies, offering a comprehensive overview of prevailing perspectives within the scientific community. A key challenge highlighted is the lack of standardized thresholds, measures, and confounders, further complicated by variations in measurement methods, the urban heat island effect, and local disparities. The Distributed Lag Non‐linear Model (DLNM) has emerged over the past decade as the gold‐An standard for modeling heat‐attributable health burdens. This approach enables risk stratification based on socio‐demographic factors, evaluation of confounding variables, and scenario simulations. Numerous adaptation strategies are proposed, and with the support of emerging technologies, scientific research is increasingly shifting toward data‐driven methodologies, unlocking new possibilities.

## Graphical abstract

This review aims to consolidate current knowledge about the public health impact of heat. Pathophysiological mechanisms are well‐known, but an ‘environmental epidemiology’ perspective is essential to drive adaptation strategies.

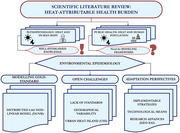

The main contents of this analysis are:

1. The current gold standard for risk assessment (DLNM)
2. Adaptation strategies, with supporting technologies
3. Critical open challenges and future perspectives

## 1 Introduction

### 1.1 Climate Change and Human Health

Long‐standing projections indicate that the global average temperature of the Earth could rise between 1.4 and 5.8 °C by the end of the current century.^[^ ^1^ ^]^ As of 2021, global temperatures had already increased by over 1.2 °C above preindustrial levels, approaching the critical threshold of keeping warming “well below 2 °C”.^[^ ^2^ ^]^ Specifically, the global land‐surface air temperature has risen by 1.53 °C since the preindustrial era (1850–1900).^[^ ^3^ ^]^ In this context, the frequency, intensity, and duration of extreme heat events are projected to continue increasing throughout the twenty‐first century, leading to record‐breaking temperatures worldwide,^[^ ^3^ ^]^ with 2022 registering the hottest summer record in Europe.^[^ ^4^ ^]^ In 2019 alone, an estimated 475 million additional individuals, primarily from vulnerable populations, were exposed to heatwave events globally.^[^ ^2^ ^]^ Although the extent to which climate change will increase the frequency and severity of extreme summer seasons remains under investigation,^[^ ^5^ ^]^ current evidence underscores the urgency of reassessing and strengthening public health adaptation and resilience strategies^[^ ^6^ ^]^ in response to the escalating risk posed by extreme heat to the biosphere, including human society,^[^ ^7^ ^]^ as also recognized by the World Health Organization (WHO).^[^ ^8^ ^]^ It was assessed that heat exposure increased by 57% between the first and the second decade of the twenty‐first century, with a corresponding rise in heat‐related mortality.^[^ ^4^ ^]^ Across all climate change scenarios, the health risks associated with extreme heat are expected to intensify significantly. There is compelling evidence that total heat‐related mortality is increasing due to climate change,^[^ ^9^ ^]^ with one third of cases attributed to anthropogenic factors.^[^ ^10^ ^]^

A wide range of diseases are influenced by climate variability, including cardiovascular mortality and respiratory illnesses exacerbated by heatwaves, as well as mental health disorders.^[^ ^11^, ^12^, ^13^ ^]^ Furthermore, climate change affects the transmission of infectious diseases and contributes to malnutrition due to crop failures.^[^ ^1^ ^]^ The relationship between extreme heat exposure and increased emergency room visits and hospitalizations is well established.^[^ ^14^ ^]^ Health‐related risks are not confined to extreme events such as heatwaves but also arise during moderately warm periods.^[^ ^15^ ^]^ Exposure to high temperatures results in a spectrum of negative health impacts,^[^ ^2^ ^]^ contributing to conditions such as heat stroke, acute cardiovascular conditions, and renal diseases.^[^ ^16^, ^17^ ^]^ The incidence of these conditions varies based on individual exposure, location, and susceptibility.^[^ ^3^ ^]^ A comprehensive analysis of the pathophysiological mechanisms underlying heat‐related health impacts is provided by Bell,^[^ ^14^ ^]^ elaborating on Sorensen et al.^[^ ^18^ ^]^ Acute heat‐related conditions include heat rash, cramps, edema, syncope, exhaustion, and stroke,^[^ ^14^ ^]^ with both internal factors (susceptibility) and external factors (vulnerability) influencing possible outcomes.^[^ ^14^ ^]^ The pathophysiological mechanisms leading to adverse health events include obstruction of eccrine sweat glands, sweating‐induced dehydration, electrolyte imbalances,^[^ ^14^ ^]^ elevated heart and breathing rates, vasodilatation, and increased or decreased coagulation.^[^ ^19^ ^]^ In response to heavy heat loads resulting from exogenous heat, endogenous heat, or both, the thermoregulatory ability of the human body may become strained or overwhelmed.^[^ ^18^ ^]^ This condition triggers a cascade of physiologic abnormalities caused by a failure to dissipate excessive body heat, including a decrease in central venous pressure, the onset of cellular and organ dysfunction, injury to the gastrointestinal tract and resulting endotoxemia, which may cause a systemic inflammatory response and a further elevation of the core body temperature.^[^ ^20^ ^]^ Additional details on these mechanisms are available in.^[^ ^21^, ^22^, ^23^, ^24^ ^]^ It is worth highlighting that cardiovascular health is particularly susceptible to the effects of climate change, both directly and indirectly. A systematic review published in 2022 provides an extensive discussion of these impacts.^[^ ^25^ ^]^

### 1.2 Climate Change as a Public Health Challenge

Despite extensive research on climate adaptation, real‐world implementation of possible counteracting strategies has been slow, frequently short‐falling,^[^ ^26^ ^]^ and is often lacking a necessary long‐term perspective.^[^ ^27^ ^]^ Although the health risks posed by climate change are well‐documented, the issue is not consistently prioritized within public health, and it is not widely regarded as an immediate concern by policymakers.^[^ ^3^ ^]^ This shortcoming partially arises from the initial framing of climate change as an environmental issue rather than a multifaceted societal challenge.^[^ ^3^ ^]^ Consequently, current adaptation efforts remain fragmented and insufficient.^[^ ^26^ ^]^ Moreover, past attempts to implement effective early adaptation responses, including preparedness and response strategies, intervention actions, and heat‐health early warning systems, have been largely insufficient to prevent significant heat‐related mortality.^[^ ^6^ ^]^ As a result, policies and strategies to effectively mitigate the adverse health impacts of temperature variability are currently lacking.^[^ ^28^ ^]^ Public health interventions play a critical role in reducing the effects of heatwaves, with scientific evidence indicating that effective public health planning could significantly reduce heat‐related fatalities under future climate scenarios.^[^ ^29^ ^]^ Scientific research suggests that healthcare systems must prepare for increased demand during warm seasons and sustained high demand during cold seasons, particularly under high‐emission climate scenarios.^[^ ^30^ ^]^ Although some evidence of adaptation to rising temperatures exists in high‐income countries, projections indicate that, without investment in research and risk management, heat‐related morbidity and mortality will likely continue to increase.^[^ ^4^, ^31^ ^]^ Investments in public health research on climate change, including monitoring and surveillance, are essential for assessing needs and identifying potential health co‐benefits of climate adaptation at both local and national levels.^[^ ^9^, ^31^ ^]^ Climate mitigation and adaptation strategies designed to prioritize health, well‐being, and equity could represent one of the most significant public health policy opportunities of the century.^[^ ^4^ ^]^ The World Meteorological Organization (WMO) states that “the health sector needs to ensure that climate information and services inform national assessment and policies”.^[^ ^32^ ^]^ However, these efforts alone are insufficient. Climate vulnerability is not solely a physical phenomenon but also a social one, requiring a long‐term, holistic approach^[^ ^26^ ^]^ in planning. A supportive political context is crucial, requiring engagement from key actors and institutions across society to fully integrate the health dimensions of climate change into policy frameworks.^[^ ^4^ ^]^ In particular, modeling frameworks are essential tools for designing national and local health and climate policies, as well as for projecting the health impacts of temperature extremes under future climatic and socioeconomic scenarios.^[^ ^33^ ^]^

### 1.3 Study Rationale

In light of this complex and concerning scenario, scientific production on climate change and health has significantly increased since the early 2000s, with a notable surge in the past seven years.^[^ ^4^ ^]^ The pathophysiological mechanisms of heat‐related health effects have been extensively studied and are now well‐established.^[^ ^14^ ^]^ However, this knowledge alone is insufficient to address the deteriorating conditions driven by climate change, highlighting the increasing need for adaptation strategies.^[^ ^34^ ^]^ A good example is the impact of heat on mental health. While the precise pathological mechanisms remain unclear (although some hypotheses have been proposed, as reported by Liu^[^ ^12^ ^]^), with a wider perspective, it is possible to identify a significant association, as demonstrated by two systematic reviews and meta‐analysis published in 2018^[^ ^11^ ^]^ and 2021.^[^ ^12^ ^]^ As previously discussed, a wider public‐health perspective is essential to generate information on an ecological, population‐scale level, that could support, with evidence‐based knowledge, the decisions of policymakers. Achieving this goal requires an inherently multidisciplinary approach that is defined as “environmental epidemiology”. It is a complex and manifold field, embracing multiple disciplines from different backgrounds, including (but not limited to):

1. Health sciences: epidemiology, medical and bio‐statistics, public health, occupational health;
2. Geomatics: Geographic Information Systems, remote sensing and Earth observation, spatial statistics and geospatial modelling, cartography;
3. Environmental sciences: meteorology and climate science, atmospheric and air quality research, exposure science;
4. Data science: data mining, big data analytics, artificial intelligence (machine learning);
5. Social and applied sciences: urban planning, sociology, risk assessment, policy analysis.

Therefore, the scope of this study is to review the most relevant scientific literature on public health studies addressing the impact of heat on population‐scale human health, to highlight available knowledge inferred from environmental epidemiology analyses, proposed technical solutions, open issues, and research directions advocated for the future. The broad scope of this study distinguishes it from existing up‐to‐date reviews, which typically focus on specific aspects of this multifaceted field. The volume of scientific literature on this topic is substantial. For instance, as early as 2018, a general‐purpose systematic review identified 188 studies.^[^ ^35^ ^]^ Furthermore, the research output in this field exponentially increased in recent years^[^ ^4^ ^]^: for instance, a search on Google Scholar with the string “environmental epidemiology health impact of heat” is returning 31 900 results published in 2024 alone. In this context, conducting a rigorous systematic review would only be feasible for specific subtopics, whereas a comprehensive review of the entire field necessitates a different approach. Consequently, the selection of studies included in this work was performed manually. The general literature screening was performed using Scopus and Google Scholar, using the keywords “heat”, “heatwave”, “health”, “impact”, “risk assessment”. Additional studies were included based on references cited in the analyzed works. The inclusion/exclusion criteria were based on content, publication date, and achieved impact. Although not all the studies addressing the topic were included, this approach aimed at depicting a comprehensive perspective of the current state‐of‐art principal stream within the scientific community. The goal was to produce a report for the widest possible audience, aiming at a better understanding of how contemporary science is approaching the problem, as well as providing a complete and organized reference body for researchers approaching this field. To achieve these objectives, a consistent schematization effort was undertaken across all sections, including some graphical representations.

**Figure 1:**
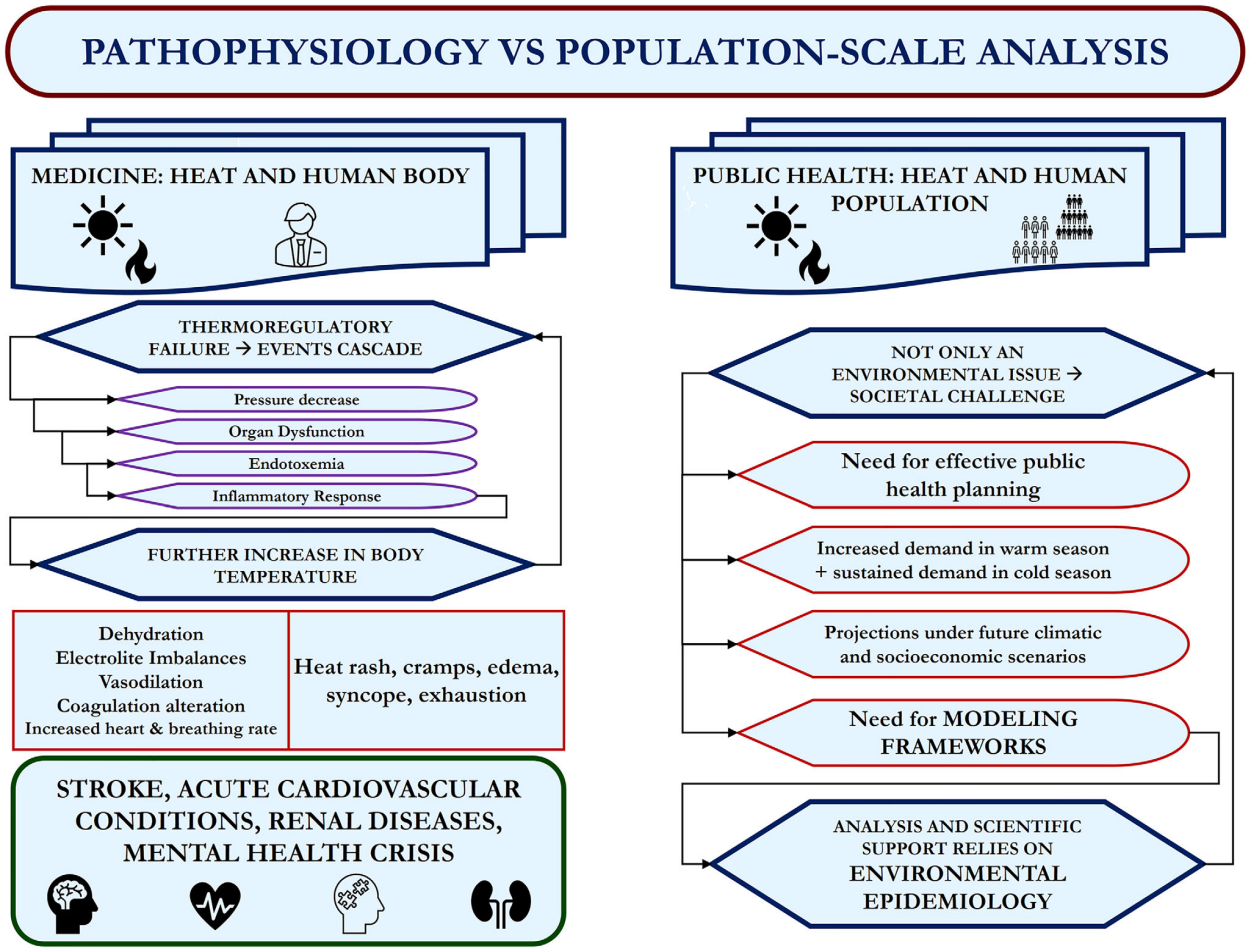
Background of the study, highlighting two parallel approaches. On the left, the core knowledge about the pathophysiological mechanisms of heat impact on the human body is summarized. On the right, issues relevant to the impact of heat on a population‐scale level are listed, suggesting the potential role of methodological approaches such as environmental epidemiology to tackle these issues with a public health perspective.

An overview of the study background is provided in **Figure** **1**. The structure of the manuscript is organized as follows: Section 2) presents methodological solutions adopted to assess the risk and to study the relationship between heat and human health at population scale, along with the obtained results and the different identified sub‐topics, building up the core content of this narrative review; Section 3) deals with some of the proposed solutions for adaptation, along with enabling new technologies; Section 4) lists different challenges and issues that still constitute a research gap, including measurements, definitions, and geographic disparities; Section 5) draws synthetical conclusions.

## 2 Current Knowledge About Heat Impact on Public Health

This work is intended to depict the current state‐of‐art about the effects of heat on human health with a public health perspective, and not focusing on the individual. Therefore, the technical means and the methodology to be applied require an environmental epidemiology approach. In order to study the relationship between any environmental stressor and related public health outcomes on a population‐scale level, it is necessary to develop a statistical model capable of representing such a relationship, allowing to quantitatively assess the risk associated with the exposure factor. Such an approach is non‐trivial, due to data availability and systematic complexities, as also recognized by the WHO.^[^ ^36^ ^]^ In the specific case of temperature as the environmental stressor, the review of existing literature clearly enlightened one modelling approach as the most widely adopted, namely the Distributed Lag Non‐linear Model (DLNM). The disproportion between the scientific literature relying on this method and that proposing any other approach (in terms of the number of studies and, more importantly, of their impact) emerged compelling, resulting in the decision to focus this review on studies adopting DLNM. At the same time, a unified description of the DLNM, encompassing all additional features and variants developed over time, appears to be currently lacking. Therefore, it was necessary to provide a comprehensive descriptive overview of this topic.

### 2.1 The Distributed Lag Non‐Linear Model

**Figure 2:**
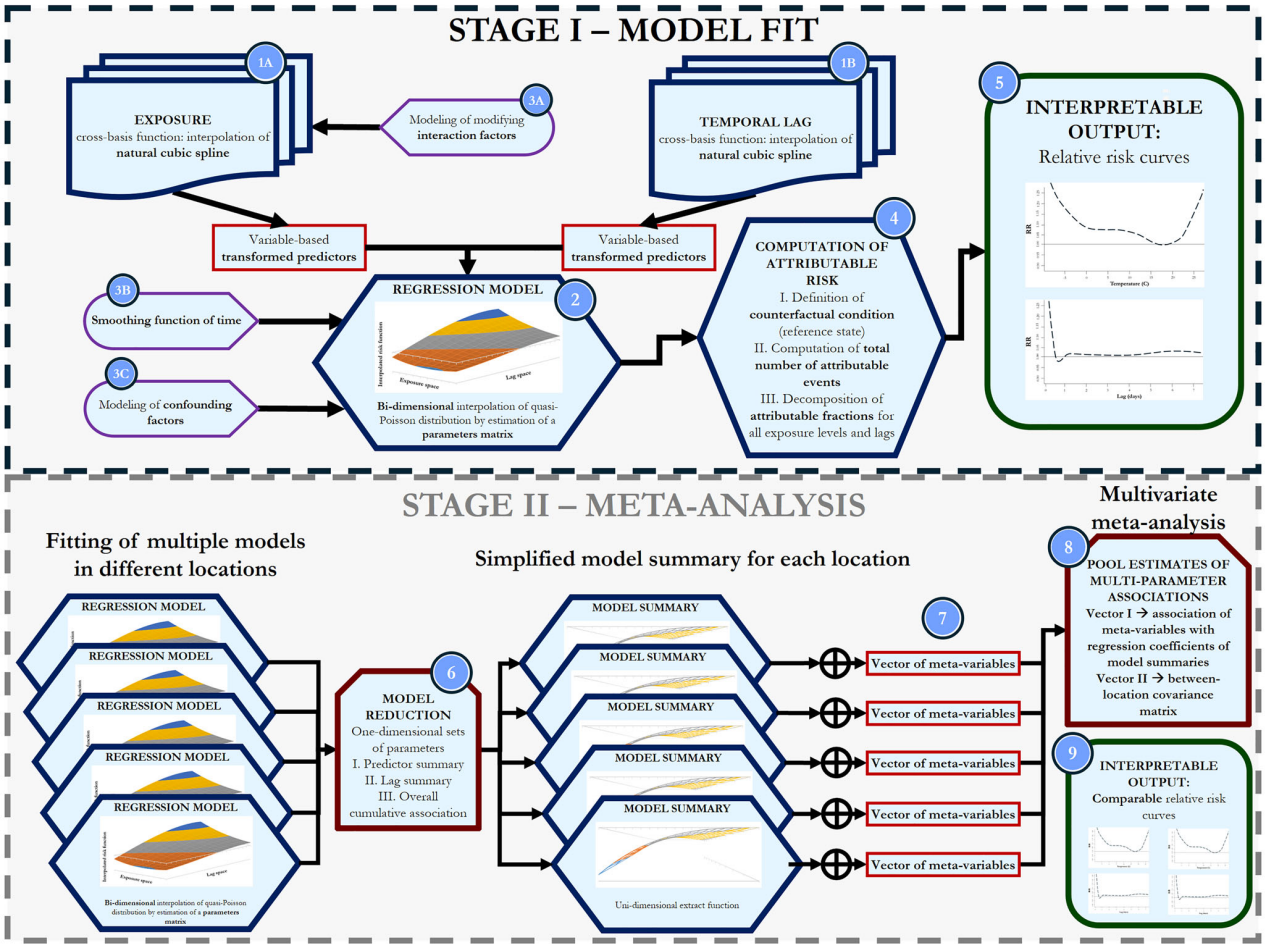
Main principles of the Distributed Lag Non‐linear Model, a statistical model to infer the relative risk function of an exposure‐outcome relationship (i.e., applied to study the short‐term impact of heat on human health). See text for detailed description of the different blocks.

The Distributed Lag Non‐linear Model (DLNM) was first proposed in 2006,^[^ ^37^ ^]^ fully formalized in 2010,^[^ ^38^ ^]^ and extended to study the health impact of heat in 2013.^[^ ^39^ ^]^ The framework is available through a package in “R” statistical environment, named “dlnm”. A graphical schematization of the main steps and functions in DLNM is reported in **Figure** **2**. The main goal of this model is to combine the effects of the intensity of exposure, modelled through a non‐linear function representing the exposure‐outcome relationship, with an additional time dimension to consider delayed effects (distributed lag). The model achieves this through a regression (Figure 2 – block 2) that assumes a quasi‐Poisson distribution for the outcome, using a “cross‐basis function” that combines two “basis functions” in a 2D space: the first defining the outcome as function of exposure intensity, and the second as a function of temporal lag from the exposure, thus allowing the model to simultaneously represent these two aspects. Both “basis functions” are obtained by fitting the data with natural cubic splines with varying degrees of freedom to control the smoothness and flexibility of the model (Figure 2 – blocks 1A‐1B), projecting the independent variables into a new space where their relationship with the outcome can be modeled linearly. This approach enables the model to flexibly capture both non‐linear relationships and lag effects through linear combinations of these transformed “predictors”, derived from the original variables and subsequently used in the regression model (Figure 2 – block 2), where their relationship with the outcome is described by a matrix of estimated coefficients. Confidence intervals are also often computed, usually through Monte Carlo simulations.^[^ ^40^ ^]^

In modelling the effect of exposure on the outcome, it is also possible to include interaction effects by incorporating additional terms into the basis function and capturing the modifying influence of other relevant variables (Figure 2 – block 3A). Moreover, when developing the final regression model, two additional components are usually included: I) a smoothing function of time, typically built using natural cubic splines or, alternatively, harmonics, used to account for temporal trends at different scales (Figure 2 – block 3B); II) confounding factors, included as additional covariates, potentially with their own basis functions to linearly incorporate these effects into the regression model (Figure 2 – block 3C).

Finally, once the model is built, the “attributable risk” (Figure 2 – block 4) is computed.^[^ ^41^ ^]^ To do so, a counterfactual condition is defined as a reference state, usually represented by the absence of association or by a baseline exposure level. The total number of attributable events is then calculated by summing the contributions from all exposure levels and their associated temporal lags. This approach, thanks to its mathematical formulation, effectively allows to decompose the risk into attributable fractions representing the proportion of the outcome that could be linked to specific exposure levels across different time lags. However, interaction effects and confounding factors are not included in such a decomposition. This approach allows to account for lagged effects both from a forward (assessing future risk related to current exposure) or backward (assessing current risk related to past exposure) perspective, but may suffer from the “harvesting” hypothesis,^[^ ^42^ ^]^ which states that an extreme exposure event may significantly reduce the pool of susceptible population, thus creating a mismatch between the observed effects at later lags and the estimated risk, that may result overestimated. With this analytical framework, it is therefore possible to obtain a “relative risk (RR)” surface, whose sections provide RR curves for different exposure levels or lag times, along with the possibility to compute the cumulative overall contribution, usually considered as the main output (Figure 2 – block 5). One key parameter that can be extracted is the Minimum Mortality Temperature (MMT), identified as the point where the temperature‐attributable health relative risk is minimal.

In environmental epidemiology, a critical goal is to compare the results emerging from different experimental set‐ups, increasing robustness and inspecting into geographical variability, which is proved to consistently affect the results.^[^ ^43^ ^]^ However, this task is not straightforward within the framework of DLNM. To this scope, a specific “second stage” of analysis is usually implemented,^[^ ^44^ ^]^ based on the multivariate meta‐analysis,^[^ ^45^ ^]^ extended to pool estimates of multi‐parameter associations.^[^ ^46^ ^]^ This second stage is also made available in “R” statistical environment with a package named “mvmeta”. Applying such approach to DLNM is challenging due to the model's high dimensionality, thus raising the need to simplify DLNM into more manageable summaries (Figure 2 – block 6), represented by a reduced set of parameters in 1D functions. Usually, three mono‐dimensional summaries are considered: 1) predictor specific summary, implemented by fixing an exposure value and considering its effects over the lag periods, with relevant confidence intervals; 2) lag‐specific summary, implemented by fixing a lag value and considering the effects over exposure values, with relevant confidence intervals; 3) overall cumulative association, computed as the net effect cumulated across the whole lag period for a fixed exposure level. On the basis of these three summaries, for each experimental set‐up a k‐dimensional set of outcome parameters, assumed to follow a k‐dimensional multivariate normal distribution, is computed (in this specific case, the regression coefficients from the first stage), along with their k × k (co)variance matrix. Again, for each set‐up, a vector of meta‐variables is computed (Figure 2 – block 7), along with its Kronecker expansion matrix. The meta‐regression model (Figure 2 – block 8) is then applied to estimate two parameters vectors, one defining how the meta‐variables are associated with each of the true k first‐stage coefficients, and a second one defining the between‐location (co)variance matrix (while the within‐location covariance matrix is assumed as known). The hereby obtained transformation enables the comparison of results across different set‐ups, thus providing pooled RR curves (Figure 2 – block 9), with the main output usually being the pooled cumulative overall contribution.

### 2.2 Results from Environmental Epidemiology Analyses

#### 2.2.1 General knowledge

Mapping health burdens is a non‐trivial task, mainly due to varying vulnerability and demographic distributions.^[^ ^33^ ^]^ Nevertheless, in the last decade, numerous epidemiological studies have documented the link between high temperatures and various health outcomes, especially mortality,^[^ ^29^ ^]^ mostly relying on DLNM adapted for application with a multi‐country perspective, as presented in.^[^ ^47^, ^48^ ^]^ A complete overview of the relevant studies applying DLNM, identified in this review, is provided in **Table** **1**. From a mathematical point of view, some studies highlighted that the temperature‐mortality relationship often exhibits a “V‐shaped” curve, with rising risks for temperatures deviating from the optimal range (MMT).^[^ ^49^, ^50^ ^]^ Others describe the exposure‐response relationship for temperature as a “U” shape^[^ ^51^ ^]^ or “reversed‐J” shape.^[^ ^33^ ^]^ The relative risk (RR) of death increases gradually for cold temperatures below the MMT, with a steeper slope for hot temperatures, particularly in case of heart failure.^[^ ^17^ ^]^ For example, over two weeks in August 2003, there were between 22 000 and 45 000 heat‐related deaths in Europe,^[^ ^1^ ^]^ with estimates of total casualties reaching 70 000,^[^ ^52^ ^]^ and 60 000 in the summer of 2022.^[^ ^6^ ^]^ Similarly, an odds ratio of 1.28 was calculated for heat‐related mortality at the 99th percentile of daily mean temperature.^[^ ^53^ ^]^ In recent observations, 7.17% of deaths were attributed to non‐optimal temperatures^[^ ^49^ ^]^ and, for every 1000 cardiovascular deaths, 2 excess deaths were attributed to extreme hot days.^[^ ^17^ ^]^ Nevertheless, other studies highlighted how moderate heat could be responsible for a larger number of casualties due to a higher occurrence frequency,^[^ ^54^ ^]^ with estimates of nearly nine times more deaths than extreme heat.^[^ ^55^ ^]^

#### 2.2.2 Vulnerability Stratification Across Population Groups

One significant factor influencing the estimation of mortality risk is age,^[^ ^56^ ^]^ which has been extensively studied in the context of temperature‐related mortality and morbidity, particularly among the elderly population (over 65 years old). A comprehensive systematic review and meta‐analysis published in 2016^[^ ^57^ ^]^ pooled data from 18 mortality and 31 morbidity studies, revealing an increased risk among elderlies for temperature‐induced cerebrovascular, cardiovascular, diabetes, genitourinary, infectious disease, heat‐related, and respiratory outcomes. These results were further corroborated by another review published in 2019,^[^ ^56^ ^]^ analyzing 207 studies from 1980 to 2017. Older adults are particularly vulnerable due to compromised thermoregulatory systems (i.e., having a reduced physiological ability to regulate body temperature under heat stress),^[^ ^55^ ^]^ underlying pathological conditions, medications that interfere with heat dissipation, mobility issues, inadequate air conditioning in their residences,^[^ ^14^ ^]^ slow physiological adaptation and behavioral response to thermal stress, limited access to medical care, and by the fact of often living alone.^[^ ^57^ ^]^ Many studies have quantified this increased vulnerability among the elderly,^[^ ^9^, ^33^, ^58^ ^]^ with Watts^[^ ^2^ ^]^ reporting a 53.7% increase in heat‐related mortality for population over 65 in the last two decades. Huber^[^ ^59^ ^]^ noted that, whereas extreme temperatures impacted younger subpopulations more severely, individuals over 80 years old resulted at increased risk even at milder temperatures.

Beyond age, gender has also been identified as a potential factor influencing heat‐related mortality and morbidity. Achebak^[^ ^55^ ^]^ reported an increased risk for women, as confirmed by Saucy^[^ ^53^ ^]^ and Yang,^[^ ^58^ ^]^ who identified older women with lower socioeconomic status as the most fragile group. Henderson^[^ ^60^ ^]^ conducted a similar study on the Canadian heat dome of summer 2021, reporting an increased risk for the elderly and women. Ballester^[^ ^6^ ^]^ provided further insights, reporting that women face 56% more heat‐related deaths than men, with higher rates in men aged 0–64 (+41%) and 65–79 (+14%) years, and in women aged 80+ years (+27%). A different result, obtained through a meta‐analysis, was reported by Liu^[^ ^12^ ^]^ regarding heat‐attributable mental health disorders, indicating greater vulnerability in males. This finding was further confirmed for morbidity by Nori‐Sarma,^[^ ^13^ ^]^ who also identified significant differences across age groups.

On this matter, Achebak^[^ ^61^ ^]^ applied the DLNM modeling approach for 48 Spanish provinces between 1980 and 2016, to study the difference in the response among population groups, specifically for age and gender, concluding that women and the elderly (over 90 years old) are more fragile. Similarly, Saucy^[^ ^53^ ^]^ analyzed data from Zurich, Switzerland, from 2000 to 2015, fitting the DLNM with a time‐stratified case‐crossover design, where each case was matched with up to four control events in the same month and day of the week, with the goal of studying cause‐specific cardiovascular mortality across different groups. Their findings confirmed an increased temperature‐related risk of mortality for myocardial infarction and hypertension, particularly among elderly women (over 75 years old) with lower socioeconomic status. Regarding the abovementioned study by Nori‐Sarma^[^ ^13^ ^]^ with relevance to mental health morbidity, a slightly higher relative risk for men compared to women was reported. Form a methodological viewpoint, a first example of stratification of results was provided by Ballester,^[^ ^6^ ^]^ who analyzed the excess heat‐related deaths through DLNM in Europe in the summer of 2022 (June to September), considering data from 823 contiguous regions in 35 European countries, stratifying the population on the base of age and gender, confirming that women and elderlies were the most fragile groups. In fact, when data are available, the stratification can be separately performed on a high‐granularity geographical level, as showed by Gasparrini,^[^ ^62^ ^]^ where authors fitted the DLNM on 34′753 “lower super output areas” within 348 local authority districts across England and Wales, with data collected between 2000 and 2019, separately for different age groups. The authors showed that the risk increased with age and was highly heterogeneous from a geographical point of view. These results were further corroborated by Masselot,^[^ ^33^ ^]^ applying a similar analytical approach across 854 European cities, considering age as an additional linear term in meta‐regression.

#### 2.2.3 Impact of Different Measurements of Heat

Considering the impact of different aspects and definitions of “ambient temperature”, two primary contributors to heat‐related health impacts can be identified: the immediate risk associated with daily temperature levels and the cumulative risk from prolonged exposure to heat over consecutive days. The latter is hypothesized to trigger unique pathophysiological mechanisms that are not present during isolated extreme heat days.^[^ ^29^ ^]^ As a consequence, extended heatwaves significantly elevate mortality rates,^[^ ^63^ ^]^ particularly among individuals with myocardial infarction and hypertension.^[^ ^53^ ^]^ Additionally, the duration and seasonality (occurring earlier in the summer season) of heatwaves have been identified as significant risk factors.^[^ ^1^ ^]^ Watts^[^ ^2^ ^]^ documented the cardiovascular and respiratory negative impacts of record‐breaking heatwaves and wildfires in Australia, western North America, and western Europe. Zhao^[^ ^64^ ^]^ provided a temporal perspective, revealing that the ratio of heatwave‐related excess deaths to total premature deaths per warm season remained stable over 30 years, while the number of heatwave‐related excess deaths per 10 million residents per warm season declined by 7.2% per decade compared to the 30‐year average. A first example is the work published by Gasparrini^[^ ^29^ ^]^ reporting results of an attributable risk assessment (via pooled effects meta‐analysis of single DLNM models). The goal was to decompose the contribution of single heat days from sustained heat waves (multiple consecutive days of heat). Analyzing data from 108 communities in the USA from 1987 to 2000, the study found that heatwaves lasting four or more days had a discernible impact, though their magnitude was considerably lower compared to the “main” effect of single heat days (0.2%–2.8% vs 4.9%–8%, respectively). Zhao^[^ ^64^ ^]^ further investigated this finding by fitting the DLNM with data from 750 locations across 43 countries, dated 1990 to 2019, defining heatwaves as periods of two or more days with a daily mean temperature exceeding the area‐specific 95th percentile. This study estimated an overall increase in mortality of 0.94% (95% CI: 0.68–1.19) per warm season.

Night‐time temperature is another relevant measure. Despite its significance, scientific literature on the health risks associated with hot nights remains sparse.^[^ ^65^ ^]^ However, evidence suggests that morbidity and mortality risks associated with hot nights are substantial,^[^ ^1^, ^63^ ^]^ with effects persisting for weeks, depending on the cause of death.^[^ ^65^ ^]^ Notably, early summer hot nights pose a higher mortality risk compared to late summer ones, though this fact is at least partially due to the “harvesting” effect^[^ ^65^ ^]^ (discussed in 2.1). Furthermore, it is demonstrated that high nighttime temperatures are particularly impactful in urban areas.^[^ ^66^ ^]^

One main work investigating this aspect is from Murage,^[^ ^67^ ^]^ who used the DLNM to study the cardiovascular mortality linked to hot nights, identified with a threshold of daily minimum temperature exceeding 16 °C, in the area of the Greater London region, United Kingdom, with data from 1993 to 2015. Adjusting for the previous day's temperature exposure, the study confirmed that nighttime exposure significantly increased cardiovascular risk, particularly for stroke (with RR = 1.65 and 95% confidence interval = 1.27 to 2.14). These findings were corroborated by Kim,^[^ ^65^ ^]^ who analyzed data from 47 Japanese prefectures between 1973 and 2015, defining hot nights as days with either minimum temperature ≥25 °C or minimum temperature ≥95th percentile in the area, highlighting a significant excess mortality risk associated with summer hot nights, particularly in early summer.^[^ ^65^ ^]^

Another relevant target measure in heat‐related health research is temperature variability. Vicedo‐Cabrera^[^ ^68^ ^]^ applied DLNM to analyze the excess mortality in six cities with data from 1985 to 2010, considering daily average temperature, inter‐day temperature variation, and diurnal temperature range (DTR). However, in this first attempt, results related to temperature variations were inconclusive. Lee^[^ ^69^ ^]^ fit the DLNM to 445 communities in 20 different countries from 1985 to 2015, considering as a measure of temperature the DTR, determining that temperature‐related mortality was not only due to daily peaks but also caused by increased temperature ranges (0.2%–7.4%, especially in moderate and warm seasons). Wu^[^ ^28^ ^]^ further explored temperature variability, defined as the standard deviation of the average of the same and previous days’ minimum and maximum temperatures, by fitting DLNM on 730 locations across 43 countries or regions, from 2000 to 2019. The study attributed 3.4% (2.2–4.6%) of excess deaths to the increase in temperature variability.

#### 2.2.4 Interaction Factors

Investigating the role of the interaction effects among multiple environmental variables is important for a better understanding of temperature‐related impacts on health. A key example is the combined effect of air pollution and non‐optimal temperatures, opening new research directions.^[^ ^51^ ^]^ Elevated air pollution levels can exacerbate heat‐related health effects, with stronger impacts on respiratory mortality compared to cardiovascular mortality,^[^ ^70^ ^]^ especially in urban environments.^[^ ^51^ ^]^ The joint effects of high ambient temperatures and pollutants such as PM_10_, PM_2.5_, O_3_, and NO_2_ on daily mortality are well‐documented on a global scale.^[^ ^71^ ^]^ However, the interaction between heat and ambient air pollution on cause‐specific mortality is not yet fully understood, and studies remain limited to specific locations.^[^ ^53^, ^70^, ^71^ ^]^ Given the importance of understanding these interactions for effective climate adaptation strategies,^[^ ^70^ ^]^ this topic warrants further scientific attention.

Saucy^[^ ^53^ ^]^ incorporated the effect modification of air pollution and aircraft noise in the case‐crossover design for DLNM, finding a worsening effect of PM_2.5_ on heart failure. Rai^[^ ^70^ ^]^ focused on air pollution interactions using summer data from 2000 to 2018, collected in 482 locations across 24 countries. Stratifying DLNM models by pollution concentration levels (5th, 50th, and 90th percentile), allowed to observe an increase in temperature‐related mortality at high pollution levels, particularly for high O_3_ levels (8.7% – 95% CI 8.7%–8.8% increase). Stafoggia^[^ ^71^ ^]^ further corroborated this applying the DLNM design on 620 cities from 36 countries in the period 1995–2020. In this case, the interaction effect of air pollution levels was modeled through linear functions, finding significant effects for O_3_ and PM_10_, and weaker but detectable effects for PM_2.5_ and NO_2_. A different approach by Pascal^[^ ^54^ ^]^ involved stratification based on urban greenness applied to 1300 municipalities in the Paris region, France, with data from 1990 to 2015. Comparing exposure‐response curves for different levels of urban greenness, they found that in cities with at least 40% tree coverage, the relative risk at the 99th temperature percentile was 2.17 [95% C.I. 1.98–2.38], whereas areas with less than 3% tree coverage had a higher relative risk of 2.57 [95% C.I. 2.47–2.68].

#### 2.2.5 Trend and Scenario Analysis

Studies dating back to 2011^[^ ^72^ ^]^ have identified a progressive shift in the seasonality of temperature‐attributable mortality, with peak incidences moving from winter^[^ ^73^ ^]^ toward summer.^[^ ^74^ ^]^ Most of the studies indicated that the increased risk of heat‐related mortality was already evident over the past two decades,^[^ ^5^, ^59^ ^]^ and vulnerability to heat steadily increased across Europe, rising by 6% from 1990 to 2019.^[^ ^4^ ^]^ However, a minority of studies argued that no clear evidence could be drawn regarding the temporal evolution of heat‐attributable mortality.^[^ ^55^ ^]^ Regardless of past trends, long‐term estimation of heat‐attributable mortality remains a priority to assess climate change's impact on public health.^[^ ^64^ ^]^ A warming climate is expected to significantly alter mortality seasonality,^[^ ^30^ ^]^ with projections indicating an increase during warm seasons and a decrease during cold seasons, although remaining high in the latter. This stands under various climate scenarios in arid, temperate, and continental zones.^[^ ^30^ ^]^ Accordingly, by the second half of the 21st century, despite a reduction of cold‐attributable mortality (particularly relevant in urban environments),^[^ ^75^ ^],^ the increase in heat‐attributable mortality will surpass such reduction.^[^ ^49^ ^]^ Gasparrini^[^ ^76^ ^]^ conducted a trends and scenario analysis using the DLNM and meta‐analysis framework on a dataset from 1984 to 2015, covering 451 locations in 23 countries across nine global regions to model the consequences of four different climate scenarios. This study was later expanded and corroborated by Guo.^[^ ^77^ ^]^ Achebak^[^ ^55^ ^]^ used the DLNM to analyze data from 47 Spanish cities in the summer months between 1980 and 2015, to assess long term trends in heat‐related mortality. By comparing RR curves across years, the authors identified an overall decrease, possibly due to physiological and/or societal adaptation. Honda^[^ ^50^ ^]^ used DLNM on data from 47 Japanese prefectures from 1972 to 2008 to evaluate climate change effects in 2030 and 2050 under WHO‐defined scenarios, finding projections to be more robust than previous models. Similarly, Huber^[^ ^78^ ^]^ applied DLNM in 12 German cities to estimate mortality impacts under a 2 °C temperature increase. Shindell^[^ ^43^ ^]^ computed exposure‐response functions for 10 U.S. cities between 1985 and 2006 to project premature deaths under three Representative Concentration Pathways for 2100, revealing a faster increase in premature deaths with higher warming levels. Martínez‐Solanas^[^ ^49^ ^]^ fit the DLNM with data from 147 regions in 16 European countries in the period 1998–2012 to analyze temperature‐mortality associations across four climate models related to three greenhouse gas emission scenarios. Findings highlighted a decline in cold‐related mortality but a greater increase in heat‐related mortality, resulting in a negative overall balance by the second half of the century, particularly in the Mediterranean areas. Lüthi^[^ ^5^ ^]^ adopted a similar approach, training and pooling DLNMs from 748 locations across 47 countries to estimate temperature‐related mortality under 234 different future climate models. The analysis revealed an overall increase in risk across all considered regions, though with varying magnitude. A closely related study by Madaniyazi^[^ ^30^ ^]^ used data from 707 locations in 43 countries or areas, dated 1969 to 2020, to fit a DLNM, projecting excess mortality for 2000–2099 under four climate change scenarios, with a specific focus on shift in mortality seasonality and its geographical variability. de Schrijver^[^ ^59^ ^]^ published a study combining trend analysis and age stratification to analyze the impact of population aging on the temperature‐mortality relationship. A DLNM fit and meta‐analyzed on data from Swiss municipalities (1969–2017) allowed to compare the aged‐population actual scenario against an ideal scenario with no population aging. The study revealed that in such an ideal scenario the heat‐related mortality in the last decade would have been 52.7% lower. García‐León^[^ ^79^ ^]^ further corroborated these results by studying 1368 European regions. They considered age‐specific characteristics and local socioeconomic vulnerabilities, modeling age as one dimension of the exposure‐response function in the DLNM framework. The study estimated a future increase in excess mortality in 11 different climate scenarios, highlighting a largely prevalent mortality increase in the over 80 years old age group.

#### 2.2.6 Interpolation of Missing Information

More broadly, the DLNM framework can be applied to interpolate missing information in both time and space. To achieve this, several studies^[^ ^28^, ^33^, ^64^, ^79^, ^80^ ^]^ introduced a third analysis stage, in which the model derived from the multivariate meta‐regression of DLNMs is used for spatial data integration (referred to as “prediction”). This allows for the inference of temperature‐mortality association using a spatially continuous grid covering the whole globe, based on a model fitted on data from 750 locations across 43 countries, spanning various intervals in the range 1990–2020.

#### 2.2.7 DLNM as the Gold Standard

Due to its consistent and widespread application, the DLNM framework could be considered the reference gold standard for assessing the impact of ambient temperature on health,^[^ ^67^ ^]^ being also widely referenced within the WHO (regional office for Europe) 2021 report.^[^ ^81^ ^]^ For example, a 2017 systematic review and meta‐analysis^[^ ^82^ ^]^ on the effects of temperature on cardiovascular mortality required the use of DLNM as an eligibility criterion for study inclusion. Yang^[^ ^58^ ^]^ used the DLNM framework for the first multi‐city assessment of the heat‐health relationship in China, considering data from 31 locations between 2007 and 2013 to determine the optimal thresholds for heatwave definition and the most vulnerable population subgroups. Similarly, Chen^[^ ^83^ ^]^ used the DLNM framework to compare the exposure‐response function of temperature on myocardial infarction in two different time periods, 1987–2000 and 2001–2014, in the area of Augsburg, Germany. Another key application of DLNM as a gold standard is in computing the MMT and its geographical variations, as demonstrated by Yin^[^ ^84^ ^]^ and Tobias.^[^ ^85^ ^]^ Mistry^[^ ^86^ ^]^ used the DLNM as a gold standard to benchmark results obtained from different data sources, namely weather ground stations and ERA5 re‐analysis satellite data, concluding that satellite‐derived data are usable. Huang^[^ ^66^ ^]^ leveraged the DLNM framework to quantify temperature‐attributable mortality in 85 European cities between 2017 and 2019, focusing on the economic burden of climate‐related health impacts. Ballester^[^ ^87^ ^]^ applied DLNM to data from 147 contiguous regions in 16 European countries from 1998 to 2004, to assess the effects of temporal aggregation, confirming that errors increase with aggregation level and that extreme heat periods are less sensitive to this effect. Iungman^[^ ^15^ ^]^ used DLNM‐based findings to evaluate two hypothetical urban scenarios across 93 European cities with data from the summer of 2015, respectively representing I) the absence of the urban heat island effect and II) a 30% increase in urban tree coverage. This study was later expanded^[^ ^88^ ^]^ to 946 European cities to study the impact of urban configurations, traffic flow, surface urban heat island, and pollution. Finally, an innovative application of DLNM was proposed by Estoque,^[^ ^89^ ^]^ who developed an index for heat‐attributable health burdens across multiple Philippine cities. This index is based on an IPCCʼs (Intergovernmental Panel on Climate Change) conceptual framework, incorporating 3 dimensions: hazard, vulnerability, and exposure, with DLNM used to determine MMT values across different cities.

**Table 1:** Summary table of literature review about studies dealing with heat‐attributable health burdens with an environmental epidemiology approach. The table reports the main topics, main results, and related references about the use of advanced modeling to study the relationship between exposure to heat and adverse health outcomes.

Topic	Main results	Refs.
2.2.1 General knowledge	Significant impact of heat on mortality is verified – “reversed‐J” shape of exposure‐outcome relationship, with optimal point (Minimum Mortality Temperature, MMT). Relative risk (RR) curves can be obtained through Distributed‐Lag Non‐linear Model (DLNM).	[1, 6, 11, 12, 13, 14, 17, 29, 33, 47, 48, 49, 50, 51, 52, 53, 54, 55]
2.2.2 Vulnerability stratification across population groups	Risk increases with age and lower socioeconomic conditions; increased risk for females over males.	[2, 6, 9, 12, 13, 14, 17, 33, 53, 55, 56, 57, 58, 59, 60, 61, 62]
2.2.3 Impact of different measurements of heat	Prolonged heatwaves impact more than the sum of contributions from single days, hot nights significantly increase risks, as do inter‐ and intra‐daily temperature ranges; “harvesting” effect to be taken into account.	[1, 2, 28, 29, 53, 63, 64, 65, 66, 67, 68, 69]
2.2.4 Interaction factors	Air pollution, in particular PM_2.5_, is recognized to worsen heat effects through combined exposure; knowledge is still limited to specific analysis set‐ups.	[51, 53, 67, 70, 71]
2.2.5 Trends and scenarios analysis	Observed progressive shift in mortality seasonality from winter to summer. Results about over‐time changes in exposure‐mortality relationship are debated, with expected risk increase for heating and population ageing, possibly compensated by societal adaptation.	[4, 5, 30, 49, 50, 55, 59, 64, 72, 73, 74, 75, 76, 77, 78, 79]
2.2.6 Interpolation of missing information	A third analysis stage is introduced on DLNM, to perform data integration in space, inferring the temperature‐mortality association in a spatially continuous grid covering the whole globe.	[28, 33, 64, 79]
2.2.7 DLNM as the gold standard	Used for multicity and multi‐country assessment, to compare different time periods, identify heat‐defining threshold, evaluate subgroups vulnerability, validate environmental data, assess the impact of confounding factors.	[15, 58, 66, 67, 81, 82, 83, 84, 85, 86, 87, 88, 89]

## 3 Proposed Solutions

### 3.1 Adaptation Strategies

**Figure 3:**
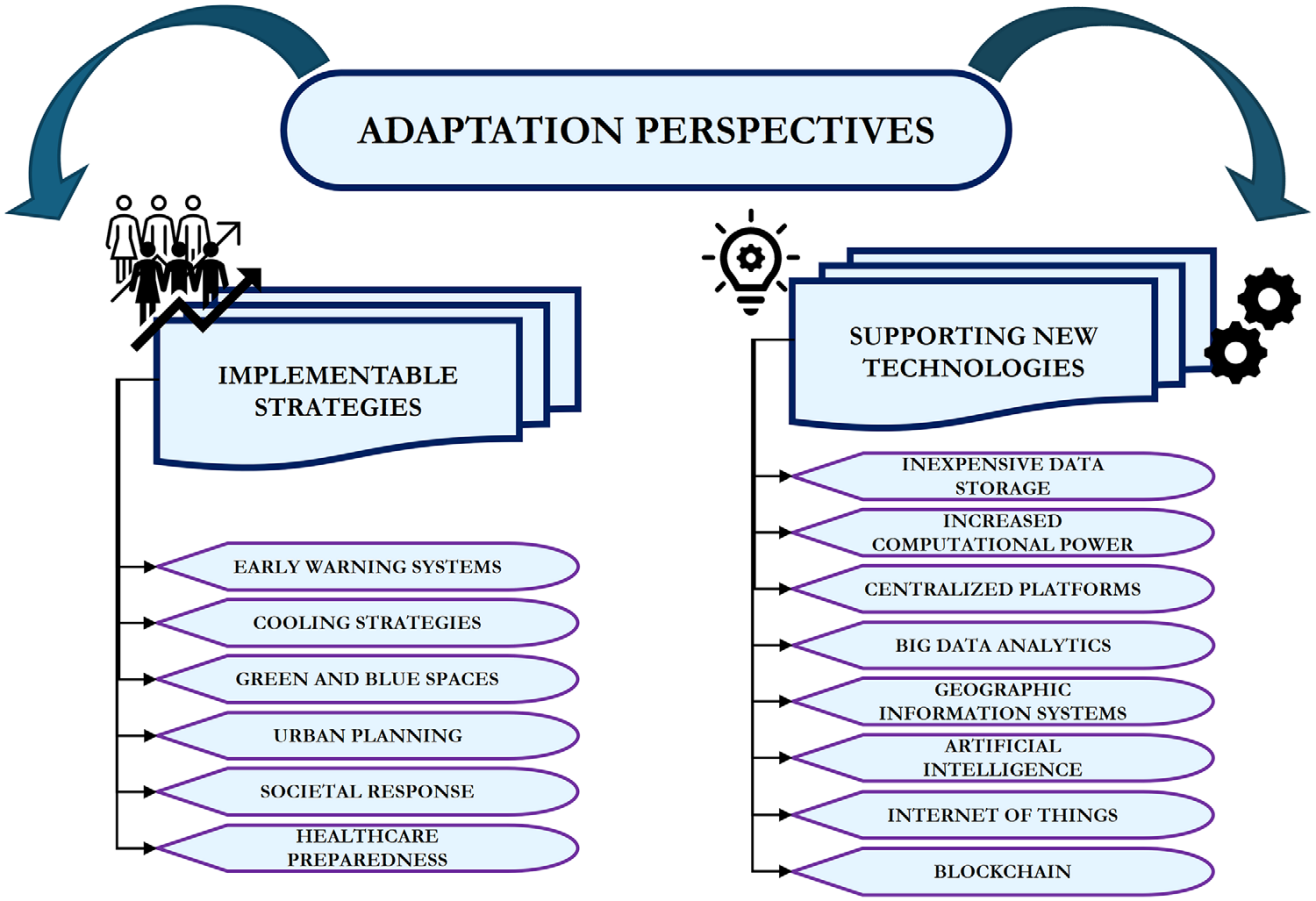
Schematic representation of the main perspectives in terms of adaptation to reduce the impact of heat on public health, considering different strategies proposed in literature (left) and technological developments supporting their implementation (right).

Based on the acquired knowledge, various adaptation strategies have been proposed to reduce the health impact of extreme heat (**Figure** **3**). A comprehensive discussion of these strategies, along with setting‐specific implementation plans, can be found in Jay.^[^ ^27^ ^]^ Across different studies, these adaptation strategies include:

I. Early warning systems:

Early warning systems (EWS) represent a cornerstone of adaptation planning,^[^ ^2^, ^9^ ^]^ as also recognized by the WHO.^[^ ^8^ ^]^ These systems help in predicting weather conditions and facilitating timely outreach to vulnerable populations, thereby preventing excessive heat exposure^[^ ^1^, ^33^, ^90^ ^]^ and reducing heat‐related fatalities ^[^ ^3^ ^]^ with increased effectiveness when accounting for temperature variability.^[^ ^28^ ^]^ A critical aspect is the accurate definition of the heatwave threshold^[^ ^58^ ^]^ at which alerts should be triggered, ideally incorporating considerations for mental health disorders.^[^ ^11^ ^]^

II. Cooling strategies:

Cooling initiatives include public cooling centers^[^ ^3^ ^]^ and stations,^[^ ^43^ ^]^ opening air‐conditioned public spaces at night for vulnerable groups,^[^ ^1^ ^]^ and improving housing conditions, particularly by increasing air conditioning use,^[^ ^89^ ^]^ especially in retirement homes, which significantly reduces heat‐related health risks.^[^ ^55^ ^]^ Henderson^[^ ^60^ ^]^ quantitatively assessed the benefits of air conditioning in lowering indoor temperatures. However, the increased reliance on air conditioning exacerbates climate change and air pollution through higher emissions,^[^ ^89^ ^]^ contributing to the urban heat island effect and therefore resulting in a non‐sustainable solution.^[^ ^2^ ^]^ Additionally, Jay^[^ ^27^ ^]^ highlighted the socioeconomic non‐sustainability of air conditioning as a long‐term adaptation strategy.

III. Nature‐Based Solutions:

Urban green spaces and infrastructures (UGI), such as green roofs and urban gardens, contribute to cooling through transpiration and shading, thereby reducing heat exposure.^[^ ^9^, ^90^, ^91^ ^]^ The expansion of tree coverage, alongside other interventions for biodiversity‐enhancing urban green spaces (e.g., parks, forests, and tree‐lined streets)^[^ ^4^ ^]^ and urban blue spaces (e.g., constructed wetlands, water plazas) can significantly lower temperatures and improve public health, particularly in cities with low cooling capabilities.^[^ ^15^ ^]^ Pascal^[^ ^54^ ^]^ quantified the protective effect of tree coverage by comparing RR among groups of municipalities, while Henderson^[^ ^60^ ^]^ found that green areas within a small geographical buffer (100 m) had a significant protective effect. Marando^[^ ^91^ ^]^ estimated that UGIs can lower urban temperatures by an average of 1.07 °C, with reductions up to 2.9 °C possible with adequate tree cover. Their study also suggested that at least 16% tree coverage is needed to achieve a 1°C urban cooling effect. Iungman^[^ ^15^ ^]^ qualitatively confirmed these findings, though with different magnitudes, estimating that increasing tree coverage to 30% could cool cities by an average of 0.4 °C, potentially preventing 2644 premature deaths annually. Labib^[^ ^92^ ^]^ further established that a 10% increase in neighborhood green space was associated with a reduction in years of potential life lost (YPLL), equivalent to an additional year of life expectancy.

IV Urban planning:

Urban configuration plays a crucial role in mitigating heat impacts, particularly regarding Urban Heat Island (UHI) intensity. Iungman^[^ ^88^ ^]^ found that green, low‐density cities exhibited significantly lower Surface UHI (SUHI) effects compared to other urban configurations. While compact cities are often considered sustainable, European compact cities face environmental and health challenges due to higher air pollution, stronger UHI effects, and reduced access to green spaces. Strategies for designing healthier cities should account for UHI seasonality, social costs, their controlling factors, and intra‐urban variability.^[^ ^66^ ^]^ Semenza^[^ ^90^ ^]^ enlightened the benefits of community‐driven initiatives such as green roofs, urban gardens, bioswales, and cool pavements to reduce heat absorption.

V. Societal response:

Community collaboration and behavioral changes, often referred to as “soft adaptation strategies”, are essential for effective heatwave response.^[^ ^90^ ^]^ They focus on altering human behavior and developing patterns to avoid risks.^[^ ^26^ ^]^ Jay^[^ ^27^ ^]^ advocated for a paradigm shift from cooling the environment to personal cooling as a more cost‐effective strategy. The understanding of personal protection behaviors linked to risk perception represents an essential step to build effective adaptation programs.^[^ ^28^ ^]^ These strategies, when integrated with biophysical solutions, constitute comprehensive heat action plans to prevent heat‐attributable health burdens.^[^ ^4^ ^]^ Key individual protective measures include limiting heat exposure, wearing appropriate clothing, staying hydrated, applying sunscreen, and using cooling devices.^[^ ^14^ ^]^ Effective interventions also involve targeted communications, wellness checks, and water distribution.^[^ ^3^ ^]^ Additionally, identifying a lead organization to coordinate preparedness, response efforts, and promoting collaboration between communities and institutions is mandatory.^[^ ^90^ ^]^ Further research is needed to evaluate the long‐term effectiveness of these interventions.^[^ ^14^ ^]^

VI. Healthcare preparedness:

Climate‐resilient health systems that integrate meteorological information are critical for risk reduction, preparedness, response, and recovery from heat events.^[^ ^3^ ^]^ Such integration is already implemented in 86 countries,^[^ ^2^, ^9^ ^]^ although geographical disparities are significant.^[^ ^32^ ^]^ As Errett^[^ ^93^ ^]^ emphasized, well‐structured public preparedness plans are essential, and strengthening healthcare resilience against heat should be a priority for public authorities through dedicated policies.^[^ ^89^ ^]^ However, the WMO enlightens how currently “data shows that the health sector is underutilizing available climate knowledge and tools”,^[^ ^32^ ^]^ thus advocating for more consistent efforts in this direction.

### 3.2 Technological Innovations

New technological developments (as reported in Figure 3) are increasingly supporting the definition and implementation of adaptation strategies. In the effort to mitigate the health impact of extreme heat, maintaining accurate records of heat‐related illnesses and deaths is essential for communities and policymakers to effectively prioritize heat‐related health programs.^[^ ^14^ ^]^ Advances in data storage and computational power have facilitated the creation of large health and environmental datasets,^[^ ^16^ ^]^ while data from population‐based cohorts could enable detailed reconstruction of individual information through database linkage.^[^ ^94^ ^]^ However, data collection capabilities vary significantly across regions, with many areas lacking surveillance systems or struggling to collect health and environmental data with sufficient completeness and granularity.^[^ ^67^ ^]^ When specific health outcome data are insufficient, natural‐cause mortality data are often used as a proxy.^[^ ^88^ ^]^ Ballester^[^ ^87^ ^]^ demonstrated that while monthly data aggregation is too coarse for estimating short‐term health effects, weekly time‐series data could provide a viable approximation for the temperature and mortality relationship on a daily scale. Contemporary technologies are opening new scenarios,^[^ ^16^ ^]^ particularly through low‐cost sensors for data collection.^[^ ^67^ ^]^ Artificial intelligence (AI), Blockchain, and the Internet of things (IoT) facilitate data integration from multiple health and environmental sources into centralized platforms^[^ ^16^ ^]^ that can support researchers, policymakers, and health officials in generating insights and applying data‐driven solutions at institutional and community levels.^[^ ^16^ ^]^ Given the availability of such structured datasets, advanced analytical techniques for big data analysis can address inconsistencies in modeling approaches, parameterization, and result interpretation across different studies.^[^ ^33^ ^]^ Machine Learning (ML) algorithms integrated with sophisticated atmospheric and climate models have significantly enhanced traditional exposure assessment methods, boosting both performance and reliability.^[^ ^94^ ^]^ ML and big data approaches are instrumental in detecting patterns within geospatial data, addressing challenges related to heat and land use.^[^ ^16^ ^]^ The geospatial component is crucial because it uniquely incorporates location‐specific social, economic, and environmental information, which is an essential element for effective policy making.^[^ ^67^ ^]^ For example, geospatial data and spatial analytics have been used to model and map metrics such as green space availability and accessibility,^[^ ^92^ ^]^ whereas mortality risk and excess deaths can be geographically visualized to highlight high‐risk areas and illustrate health disparities.^[^ ^33^ ^]^ Moreover, Geographic Information Systems (GIS) play an important role in creating comprehensive environmental databases, linking individual‐level exposures to population‐based cohorts using high‐resolution spatial‐temporal data.^[^ ^94^ ^]^

## 4 Open Challenges

### 4.1 Heat Measurement and Definitions

Even small temperature changes at mild or moderate levels can significantly affect health due to their high frequency of occurrence.^[^ ^15^ ^]^ Therefore, accurate and representative temperature measurements are critical. However, several issues remain that require further research and standardization:

I. Measurement devices. Epidemiological studies on health risks induced by non‐optimal temperatures typically rely on ground‐based weather station observations.^[^ ^86^ ^]^ The increasing capacity of cloud storage has facilitated continuous data collection via portable sensors.^[^ ^16^ ^]^ Nonetheless, ground measurements often suffer from limited spatial and temporal coverage.^[^ ^86^ ^]^ Using fine‐scale spatial‐temporal temperature data could instead help mitigating exposure misclassification.^[^ ^53^ ^]^ Moreover, exposure assessments based on ground stations may be compromised by discrepancies between outdoor temperatures (recorded by stations) and indoor temperatures, where people spend most of their time.^[^ ^11^ ^]^ This is also sensitive to the specific target population, with the measurement strategy to be possibly tailored consequently (e.g., workplace measurements for work‐related heat stress^[^ ^97^ ^]^ or indoor air temperature for maternal and newborn health^[^ ^36^ ^]^). To improve spatial detail, one potential solution is to incorporate remote sensing data^[^ ^89^ ^]^ or climate reanalysis data, which provide comprehensive spatial‐temporal exposure estimates, although their accuracy may vary, particularly in tropical regions.^[^ ^86^ ^]^ For example, land surface temperature (LST) data, despite being an indirect measure, has been proven effective for spatially explicit temperature analyses.^[^ ^89^, ^91^ ^]^ Land‐use regression is another approach, capable of modelling city‐wide outdoor and indoor air temperatures by incorporating factors such as vegetation, impervious surfaces, and building characteristics.^[^ ^63^ ^]^

II. Definition of heat. Regardless of the measurement method, how to define a condition of heat is critical, due to a lack of standards and agreement on definitions, as also acknowledged by the WHO.^[^ ^36^ ^]^ A recent systematic review^[^ ^95^ ^]^ on heat's impact on cardiovascular health highlighted an extreme heterogeneity in applied definitions, with 21 identified different combinations of criteria and varying threshold values, despite long‐standing recognition of this issue.^[^ ^96^ ^]^ The concept of a “heatwave” similarly lacks standardization,^[^ ^81^ ^]^ with definitions varying based on temperature thresholds and durations.^[^ ^14^, ^29^ ^]^ International bodies such as the WMO, the WHO, and the IPCC have proposed some definitions of “heatwave”,^[^ ^98^ ^]^ yet remaining on a very general level (e.g., “Marked warming of the air […] over a large area; it usually lasts from a few days to a few weeks”; “a period of abnormally hot weather, often defined with reference to a relative temperature threshold, lasting from two days to months”; “a period of marked and unusually hot weather persisting for at least two consecutive days”), not suitable for a rigorous scientific assessment. Some definitions incorporate not only sequences of extremely hot days but also periods where sustained heat leads to excess mortality beyond daily temperature contributions.^[^ ^29^ ^]^ For example, one definition sets a heatwave as periods lasting at least two days with location‐specific daily mean temperatures at or above the 95th percentile of the annual temperature range.^[^ ^64^ ^]^ Yang^[^ ^58^ ^]^ proposed testing multiple thresholds (for both temperature and duration) and selecting those that maximize model fitting (e.g., 92.5th temperature percentile and a duration exceeding three days^[^ ^58^ ^]^).

III. Measured value. An additional source of variability is represented by the specific temperature metric used. For example, the WMO and the WHO suggest multiple ways of computing temperature metrics that are specifically relevant for health assessment, such as wet‐bulb globe temperature (accounting for temperature, humidity, wind speed, and thermal radiation), the universal thermal climate index (accounting for air temperature, wind velocity, humidity, and mean radiant temperature), or the more general heat index (computable through five different formulas combining air temperature and relative humidity).^[^ ^97^ ^]^ This issue is furtherly complicated by the limited willingness of countries, recognized by WHO, to add new measurements to those routinely collected.^[^ ^36^ ^]^ Furthermore, once the measurement method for the temperature metric is defined, multiple options arise to define daily values, including daily maximum or minimum temperature, derived from hourly measurements, or the mean temperature (i.e., the average of these values).^[^ ^29^ ^]^ Alternatively, temperature variability can be considered, often calculated as the standard deviation of minimum and maximum temperatures over the current and previous days,^[^ ^28^ ^]^ or as the diurnal temperature range, representing the difference between day and night temperatures.^[^ ^66^ ^]^ Other measures include hot nights, defined as days when the minimum temperature is 25 °C or higher.^[^ ^65^ ^]^

In conclusion, when conducting epidemiological studies on heat's impact on human health, it is essential to carefully define the experimental set up in terms of temperature measurement and definition of heat conditions. Despite extensive scientific literature, a universally accepted definition of heat‐related death is still missing.^[^ ^14^ ^]^ This lack of standardization hinders effective comparisons and the feasibility of a meta‐analytical approach across studies. While ongoing scientific research can help address these issues, coordinated efforts by international scientific bodies are strongly needed.

### 4.2 Urban Heat Island

One specific topic frequently examined in the field is the urban heat island (UHI) effect, comprehensively discussed in Stewart & Oke.^[^ ^99^ ^]^ This concept is well‐known since the early 20th century,^[^ ^1^ ^]^ and first documented in 1947.^[^ ^99^ ^]^ It is defined as the occurrence of higher temperatures in urban areas compared to their rural surroundings. It results from several factors, such as reduced evaporative cooling, increased heat storage, and enhanced sensible heat flux due to diminished vegetation cover and the greater heat retention of buildings and paved surfaces.^[^ ^1^, ^9^ ^]^ Indeed, buildings, roads, and other infrastructures absorb and re‐emit solar heat more than natural landscapes like forests and water bodies.^[^ ^4^ ^]^ Moreover, urban morphology contributes by increasing the multiple scattering of shortwave radiation and trapping longwave radiation, thereby intensifying heat storage in cities.^[^ ^91^ ^]^ The UHI effect is significant not only when comparing urbanized areas to their rural surroundings, but also within different districts in the same city, creating varying levels of vulnerability.^[^ ^100^ ^]^ Climate change is expected to exacerbate UHI intensity, leading to higher urban temperatures and increased heat‐related health risks,^[^ ^15^, ^91^ ^]^ further compounded by the expansion of the built environment.^[^ ^15^ ^]^ For instance, Heaviside^[^ ^101^ ^]^ found that UHI contributed to almost 50% of heat‐attributable mortality in the 2003 heatwave in UK's West Midlands. This poses additional challenges for environmental epidemiology, with the need to accurately measure the UHI effect and incorporate it into risk assessments. However, different viewpoints exist regarding UHI. Some studies have found that UHI effects are particularly pronounced during heat extremes, with a median 45% increase in mortality risk reported by Huang,^[^ ^66^ ^]^ whereas others noted that night‐time UHI effects are significantly greater than daytime effects, further contributing to health risks.^[^ ^15^ ^]^ Measurements methods also vary. The extent and distribution of UHI can be estimated using air temperature and LST data.^[^ ^91^ ^]^ Although air temperature measurements provide continuous and representative UHI data, they require a sufficient number of weather stations,^[^ ^91^ ^]^ a condition often unmet in city centers.^[^ ^100^ ^]^ Various methods exist for measuring SUHI, and this parameter is influenced by factors such as urban configuration, geographical location, and climatic conditions.^[^ ^88^ ^]^ These methods often require additional computational and processing efforts, which can introduce uncertainty. The number of heat island studies has increased significantly in recent decades, underscoring the importance of expanding the research in this area,^[^ ^99^ ^]^ as this is critical to guide interventions through collaboration between communities and institutions, supported by timely meteorological forecasts and community outreach to vulnerable groups.^[^ ^90^ ^]^ However, the issue remains unresolved, and the scientific community advocates that future studies should incorporate greater granularity, considering social vulnerabilities, individual indoor/outdoor exposures, and population mobility to capture the spatial and temporal heterogeneities in UHI exposure and health risks.^[^ ^66^ ^]^

### 4.3 Geographical Disparities in Vulnerability

As discussed in Section 2.2.2, and confirmed by the WMO,^[^ ^32^ ^]^ several studies demonstrated that different population groups exhibited varying levels of vulnerability, strongly influenced by demographic distribution.^[^ ^33^ ^]^ Spatial differences in vulnerability arise from socioeconomic, cultural, and health‐related factors.^[^ ^61^, ^64^ ^]^ On a local scale, additional elements such as the UHI effect, healthcare access, and land cover further influence susceptibility to heat.^[^ ^33^ ^]^ Factors like poverty, inequality, and marginalization further burden vulnerable communities facing a worsening climate.^[^ ^26^ ^]^ Consequently, groups such as the elderly, the poor, individuals with pre‐existing medical conditions,^[^ ^19^, ^63^ ^]^ as well as people with disabilities, outdoor workers, and residents in marginal areas^[^ ^2^ ^]^ are particularly susceptible to heat‐related health risks. It is important to note that social disparities affect not only the inherent vulnerability of a population (its propensity to suffer damage when exposed to a hazard), but also the level of exposure (defined as the intersection of the spatial distribution of human populations with a hazard) in the case of ambient temperature, particularly in urban environments.^[^ ^102^ ^]^ Given the nature of these influencing factors, both vulnerability and exposure discrepancies are inherently location‐dependent.

Geographical differences in mortality risks are well documented,^[^ ^33^ ^]^ including temporal variations in heatwave‐related mortality burdens.^[^ ^64^ ^]^ Regional analyses indicate that the threshold for heat‐induced hospitalizations varies, underscoring the need for localized health strategies.^[^ ^14^ ^]^ Implementing adaptation policies is especially beneficial in regions where an imbalance exists between declining cold‐ and increasing heat‐attributable mortality.^[^ ^49^ ^]^ Recent studies report that, over the past 30 years, heatwave‐related excess death ratios have been the highest in polar and alpine climates and the lowest in tropical regions.^[^ ^64^ ^]^ Moreover, the health impacts of climate change are disproportionately affecting those who contributed the least to global warming.^[^ ^2^ ^]^ Despite an overall warming trend, the frequency, intensity, and duration of heatwaves vary both within and across countries.^[^ ^64^ ^]^ A systematic review^[^ ^35^ ^]^ revealed that most of the scientific production on this topic focuses on mid‐latitude, high‐income countries with low‐ to medium‐population densities, areas that do not correspond to those expected to experience the most extreme heatwaves in the future. Low‐ and medium‐income countries remain underrepresented, despite the need for location‐specific research to capture unique spatiotemporal characteristics.^[^ ^67^ ^]^ Consequently, regional disparities in heatwave‐related mortality have not been fully assessed globally, which hinders localized adaptation planning and effective risk management.^[^ ^64^ ^]^ In light of the non‐linear effects of new climatic extremes and varying community vulnerabilities, significant health impacts are anticipated without the implementation of adequate adaptation measures.^[^ ^5^ ^]^

Scientific research has also highlighted that Europe has emerged as a major hotspot, as confirmed by the European regional office of WHO,^[^ ^81^ ^]^ with notable increases in both seasonal averages and climate extremes.^[^ ^49^ ^]^ The average surface air temperature in Europe has risen almost 1 °C more than the global average.^[^ ^4^ ^]^ In particular, the Euro‐Mediterranean region is considered to be a significant climate change hotspot due to global warming.^[^ ^55^ ^]^ While cold‐attributable mortality remains relatively uniform across European regions, heat‐attributable mortality is significantly higher. This regional vulnerability underscores the pressing need for effective adaptation and mitigation strategies, as emphasized by the European scientific community.^[^ ^6^ ^]^. For instance, in 2021 Italy led with 66 publications on the topic, followed by Spain with 65 and Germany with 47.^[^ ^4^ ^]^ Unfortunately, this extensive scientific output has not effectively translated into significant interventions by public authorities, as discussed in Section 1.2.

Moreover, all Western countries are experiencing increased risk due to general demographic trends.^[^ ^55^ ^]^ Given the increased vulnerability of the elderly (as addressed in Section 2.2.2), research on the impact of population aging on historical temperature‐mortality trends appears very important.^[^ ^59^ ^]^ In this context, Zhao^[^ ^64^ ^]^ demonstrated that population aging contributes to differences in the temporal change of heatwave‐related death ratios between standardized and unstandardized scenarios, confirming its significance as a factor.

Despite the multitude of cited vulnerability factors, several studies indicated a decline in the RR of heat‐related mortality in developed countries.^[^ ^61^ ^]^ The alignment of rising minimum mortality temperatures with annual mean temperatures suggested that changes in the exposure–response curves, rather than warming per se, are driving the evolution of heat‐ and cold‐attributable mortality.^[^ ^61^ ^]^ Population adaptation to local climates is a key element in understanding the variability in heat‐related mortality.^[^ ^29^ ^]^ An “acclimatization” effect has been hypothesized, with later summer months showing lower risks compared to earlier ones.^[^ ^1^, ^9^ ^]^ For example, Achebak^[^ ^61^ ^]^ found that simultaneous increases in annual mean temperature and minimum mortality temperature in Spain indicated that reduced vulnerability to heat was likely a result of acclimatization. Consequently, the authors hypothesized that societal adaptation and socioeconomic development contributed to a general decline in heat‐related mortality.^[^ ^55^ ^]^ This notion is further supported by Kim,^[^ ^65^ ^]^ who found that adaptation and acclimatization likely mitigated the effects of hot nights in warmer regions. However, it was also acknowledged that warmer areas in developed countries may be nearing the limits of societal adaptation, making this aspect particularly relevant in currently cold climates.^[^ ^43^ ^]^

**Figure 4:**
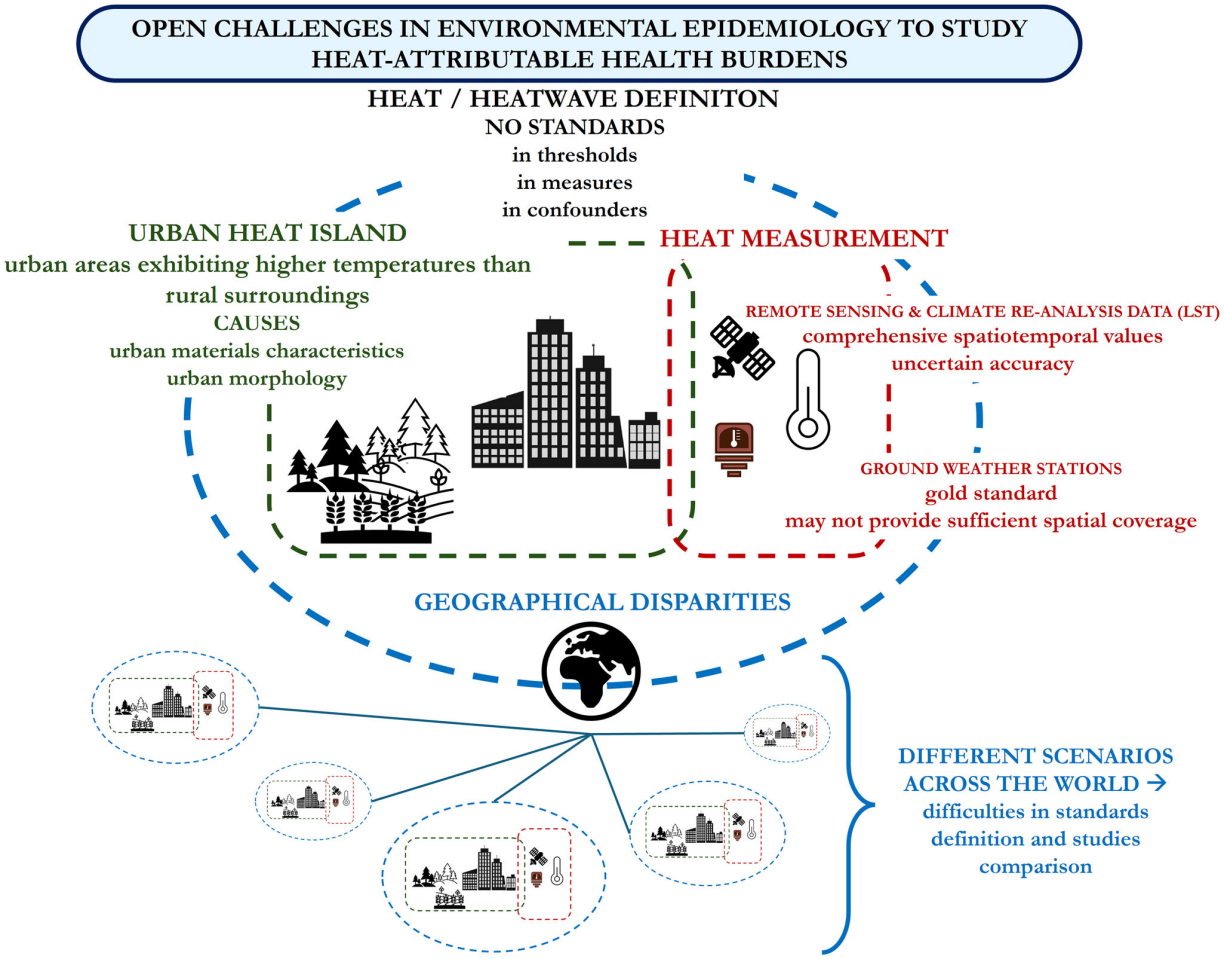
Graphical representations of the main open challenges hindering scientific research in addressing the impact of heat on public health using an environmental epidemiology approach. In particular, issues are related to the lack of universal standards (black), variety of heat measuring methods (red), urban heat island effect (green) and geographical disparities across regions (blue).

The significant discrepancies across geographical contexts exacerbate the challenges posed by the lack of standards discussed in Section 4.1, further complicating environmental epidemiology analyses. Scientists emphasized that accurately assessing risk requires an experimental set‐up that should account for location‐specific socioeconomic and environmental variables.^[^ ^33^ ^]^ Currently, no universally recognized standard exists to address geographical disparities, leaving this challenge to individual researchers. An overview of the mian current challenges in the field is reported in **Figure** **4**.

### 4.4 Future Developments

**Figure 5:**
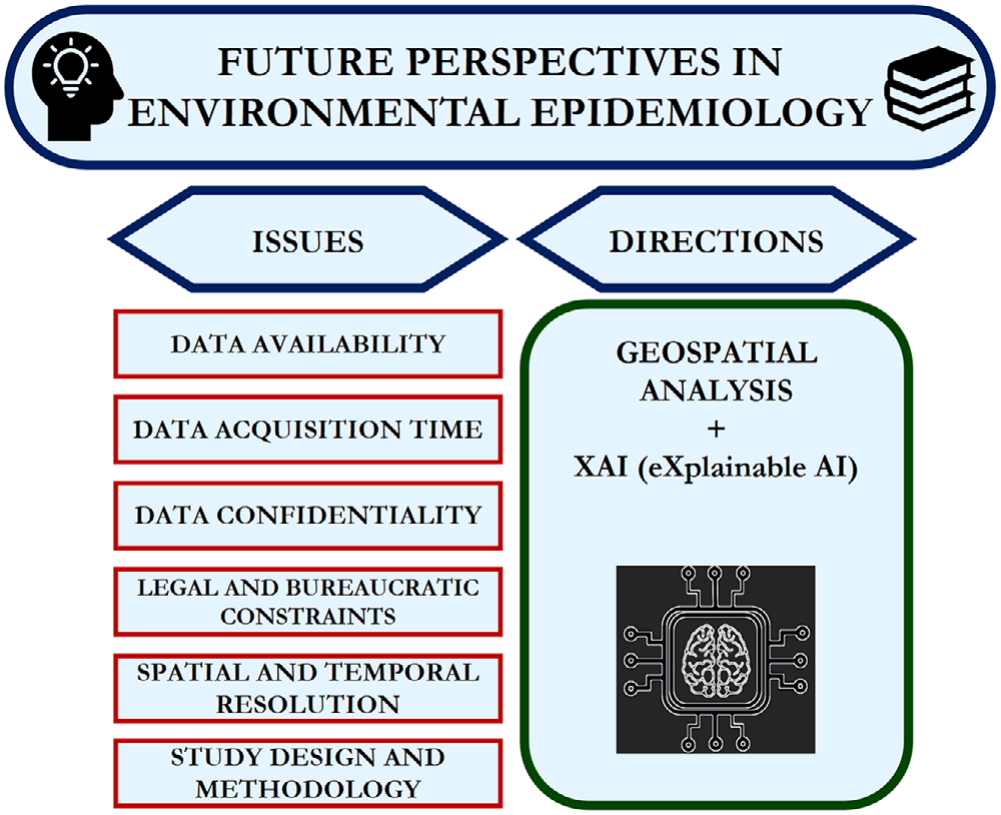
Schematization of the future directions of scientific research in environmental epidemiology in order to address heat‐attributable health burdens in a public health perspective, listing issues (left) and the most relevant developments (right).

Expanding the evidence base on the need for additional interventions could cost‐effectively reduce the health risks associated with extreme events.^[^ ^3^ ^]^ For example, focusing on morbidity rather than mortality (which is more commonly studied) may increase the sensitivity of the analyses.^[^ ^35^ ^]^ This approach has been proposed by Nori‐Sarma^[^ ^13^ ^]^ in relation to mental health in the United States, where emergency department admissions served as a morbidity indicator. However, research efforts are often constrained by issues such as confidentiality, legal constraints, data acquisition time, and bureaucratic obstacles.^[^ ^87^ ^]^ Additional challenges include limited data availability, insufficient spatial and temporal resolution, and complexities in study design and methodology.^[^ ^62^ ^]^ Moreover, current models may underestimate potential heat‐related mortality risks by failing to fully capturing a broad range of possible climate futures.^[^ ^5^ ^]^ It is also crucial to incorporate spatial autocorrelation in predictive models to enhance accuracy.^[^ ^51^ ^]^ In urban areas, multiple environmental exposures often co‐locate and cluster, thus generating interactions that may be spatially correlated.^[^ ^103^ ^]^ Ignoring these spatial patterns could lead to inaccurate health effect estimates and suboptimal predictions.^[^ ^51^ ^]^ In this context, ML algorithms such as Random Forest (RF) and Artificial Neural Networks (ANN) are well‐suited for multi‐exposure models, due to their flexibility in handling highly correlated variables and nonlinearity.^[^ ^104^ ^]^ In particular, RF models are effective in managing complex interactions among diverse variables, making them ideal for studying the influence of multiple exposures on health outcomes.^[^ ^51^ ^]^ Furthermore, the interpretation of the output of ML models, specifically the importance of independent variables and exposure‐response relationships, can be studied by applying explainable artificial intelligence (XAI) techniques.^[^ ^51^ ^]^ Methods such as permutation importance,^[^ ^105^ ^]^ partial dependence, two‐way interaction plots,^[^ ^106^ ^]^ and SHapely Additive exPlanation (SHAP)^[^ ^107^, ^108^ ^]^ could help in overcoming the “black box” nature of ML models by quantifying the relative importance of exposure variables on health outcomes.^[^ ^109^, ^110^ ^]^ Moreover, XAI approaches can generate exposure‐response curves, offering a distinct advantage over traditional methods by eliminating the need to categorize exposure variables and thus providing a more nuanced representation of sharp changes.^[^ ^111^, ^112^ ^]^ This novel analytical approach, combining XAI with big data processing and geospatial techniques, has the potential to enhance the understanding of how individual variables contribute to the overall exposure‐response relationship between heat and human health. Such insights could provide highly valuable information for policymakers to support preventive actions across multiple levels of adaptation. Despite the substantial research conducted on this topic, even for well‐studied regions such as Europe, the overall level of evidence remains insufficient, highlighting the need for further investigation by the scientific community,^[^ ^34^ ^]^ especially for the most vulnerable groups in the general population, currently marked as understudied by the WHO.^[^ ^36^ ^]^. An overview of future persepctives in the field is reported in **Figure** **5**.

## 5 Conclusion

The impact of heat on human health is an increasingly urgent concern in the context of climate change. Although the underlying pathophysiological mechanisms are well established, effective adaptation and mitigation strategies require a public health perspective. This calls for a novel approach, framed within the discipline of “environmental epidemiology”. Accordingly, this review summarized, critically analyzed, and synthesized the currently available knowledge on the relationship between heat exposure and its effects on health at ecological and population‐scale levels, thus providing a conceptual framework, identifying gaps in knowledge, and relating the findings to policy, practice, and interdisciplinary applications. Unlike previous studies that rely on systematic reviews, where the focus is narrowed to specific subtopics due to the vast number of publications, a manual evaluation of studies, based on the papers contents, publication date, and achieved impact, was performed here. This approach enabled a comprehensive overview of the current state‐of‐art, supporting its utility as a scientifically‐grounded guide for both newcomers to the field and researchers interested in specific aspects while maintaining a broad contextual perspective. An important aspect of this work was the systematic framing of the technical background, particularly regarding the DLNM. Although it has become the most widely adopted model for assessing heat impacts on public health over the past decade, its implementation has evolved progressively through stratified developments and additional variants. Therefore, collecting multiple studies and summarizing their results provided a comprehensive overview of the current DLNM state of the art, also represented by a schematic overview.

The main points arising from the examined literature revealed that:

− Heat disproportionately affects vulnerable populations, particularly the elderly, women, and socioeconomically disadvantages groups.
− Rising temperatures impact health not only through increases in maximum or average daily values but also via significant rises in nighttime temperatures and overall temperature variability (both inter‐ and intra‐daily). Moreover, prolonged heat periods have a greater impact on health than isolated heat days.
− Combined environmental stressors, in particular atmospheric pollution, exacerbate heat effects.
− Future environmental projections indicate a rising, yet uneven, impact of heat, partially balanced by increasing temperatures during cold seasons. However, studies report significant discrepancies in quantifying these changes.

Enhanced knowledge and improved evaluation capabilities have led to the proposal of many adaptation strategies. These strategies range from urban planning and infrastructure building to increased preparedness by public authorities, with a primary emphasis on “soft” strategies that foster societal awareness and behavioral responses. Technological advancements such as ICT improvements, earth observation (EO) from satellites, artificial intelligence (AI), blockchain, and the internet of things (IoT), are favoring knowledge development through scientific research and simultaneously fostering the implementation of these strategies. Nevertheless, several challenges remain. There is a lack of universally accepted standards and definitions (i.e., heat and temperature measurements), significant geographical disparities in vulnerability and adaptation levels, and persistent issues related to the UHI effect in densely urbanized areas. To further advance the understanding of these phenomena, current research is exploring new directions, particularly through the application of XAI approaches supported by big data processing and geospatial methods. These innovative tools could result in quantitative support to public authorities in planning and implementing more effective and optimized interventions.

## Conflict of Interest

The authors declare no conflict of interest.

## References

[1] J. A. Patz , D. Campbell‐Lendrum , T. Holloway , J. A. Foley , Nature 2005, 438, 310. 16292302 10.1038/nature04188

[2] N. Watts , M. Amann , N. Arnell , S. Ayeb‐Karlsson , J. Beagley , K. Belesova , M. Boykoff , P. Byass , W. Cai , D. Campbell‐Lendrum , S. Capstick , J. Chambers , S. Coleman , C. Dalin , M. Daly , N. Dasandi , S. Dasgupta , M. Davies , C. di Napoli , A. Costello , The Lancet 2021, 397, 129

[3] K. L. Ebi , J. Vanos , J. W. Baldwin , J. E. Bell , D. M. Hondula , N. A. Errett , K. Hayes , C. E. Reid , S. Saha , J. Spector , P. Berry , Annual Review of Public Health 2021, 42, 293. 10.1146/annurev-publhealth-012420-105026 PMC9013542 33406378

[4] K. R. van Daalen , M. Romanello , J. Rocklöv , J. C. Semenza , C. Tonne , A. Markandya , N. Dasandi , S. Jankin , H. Achebak , J. Ballester , H. Bechara , M. W. Callaghan , J. Chambers , S. Dasgupta , P. Drummond , Z. Farooq , O. Gasparyan , N. Gonzalez‐Reviriego , I. Hamilton , R. Lowe , The Lancet Public Health 2022, 7, 942

[5] S. Lüthi , C. Fairless , E. M. Fischer , N. Scovronick , Armstrong , M. D. S. Z. S. Coelho , Y. L. Guo , Y. Guo , Y. Honda , V. Huber , J. Kyselý , E. Lavigne , D. Royé , N. Ryti , S. Silva , A. Urban , A. Gasparrini , D. N. Bresch , A. M. Vicedo‐Cabrera , Nat. Commun. 2023, 14, 4894. 37620329 10.1038/s41467-023-40599-x PMC10449849

[6] J. Ballester , M. Quijal‐Zamorano , R. F. Méndez Turrubiates , F. Pegenaute , F. R. Herrmann , J. M. Robine , X. Basagaña , C. Tonne , J. M. Antó , H. Achebak , Nat. Med. 2023, 29, 1857. 37429922 10.1038/s41591-023-02419-z PMC10353926

[7] W. Thuiller , Nature 2007, 448, 550. 17671497 10.1038/448550a

[8] World Health Organization , The Synergies of Heat Stress and Air Pollution and its Health Impacts: Technical Brief, World Health Organization, Geneva, Switzerland 2025.

[9] N. Watts , W. N. Adger , P. Agnolucci , Environnement, Risques et Sante 2015, 14, 1861.

[10] A. M. Vicedo‐Cabrera , N. Scovronick , F. Sera , D. Royé , R. Schneider , A. Tobias , C. Astrom , Y. Guo , Y. Honda , D. M. Hondula , R. Abrutzky , S. Tong , M. D. S. Z. S. Coelho , P. H. N. Saldiva , E. Lavigne , P. M. Correa , N. V. Ortega , H. Kan , S. Osorio , J. Kyselý , A. Urban , H. Orru , E. Indermitte , J. J. K. Jaakkola , N. Ryti , M. Pascal , A. Schneider , K. Katsouyanni , E. Samoli , F. Mayvaneh , et al., Nat. Clim. Chang. 2021, 11, 492. 34221128 10.1038/s41558-021-01058-x PMC7611104

[11] R. Thompson , R. Hornigold , L. Page , T. Waite , Public Health 2018, 161, 171. 30007545 10.1016/j.puhe.2018.06.008

[12] J. Liu , B. M. Varghese , A. Hansen , J. Xiang , Y. Zhang , K. Dear , M. Gourley , T. Driscoll , G. Morgan , A. Capon , P. Bi , Environm. Int. 2021, 153, 106533. 10.1016/j.envint.2021.106533 33799230

[13] A. Nori‐Sarma , S. Sun , Y. Sun , K. R. Spangler , R. Oblath , S. Galea , J. L. Gradus , G. A. Wellenius , JAMA Psychiatry 2022, 79, 341. 35195664 10.1001/jamapsychiatry.2021.4369 PMC8867392

[14] M. L. Bell , A. Gasparrini , G. C. Benjamin , N. Engl. J. Med. 2024, 390, 1793. 38749034 10.1056/NEJMra2210769

[15] T. Iungman , M. Cirach , F. Marando , E. Pereira Barboza , S. Khomenko , P. Masselot , M. Quijal‐Zamorano , N. Mueller , A. Gasparrini , J. Urquiza , M. Heris , M. Thondoo , M. Nieuwenhuijsen , Lancet 2023, 401, 577. 36736334 10.1016/S0140-6736(22)02585-5

[16] F. M. Bublitz , A. Oetomo , K. S. Sahu , A. Kuang , L. X. Fadrique , P. E. Velmovitsky , R. M. Nobrega , P. P. Morita , Int. J. Environm. Res. Public Health 2019, 16, 3847. 10.3390/ijerph16203847 PMC6843531 31614632

[17] B. Alahmad , H. Khraishah , D. Royé , A. M. Vicedo‐Cabrera , Y. Guo , S. I. Papatheodorou , S. Achilleos , F. Acquaotta , B. Armstrong , M. L. Bell , S. C. Pan , M. de Sousa Zanotti Stagliorio Coelho , V. Colistro , T. N. Dang , D. van Dung , F. K. De’ Donato , A. Entezari , Y. L. Guo , M. Hashizume , P. Koutrakis , Circulation 2023, 147, 35. 36503273 10.1161/CIRCULATIONAHA.122.061832 PMC9794133

[18] C. Sorensen , J. Hess , N. Engl. J. Med. 2022, 387, 1404. 36170473 10.1056/NEJMcp2210623

[19] A. Peters , A. Schneider , Nat. Rev. Cardiol. 2021, 18, 1. 33169005 10.1038/s41569-020-00473-5 PMC7649889

[20] Y. Epstein , Y. R. Heatstroke , N. Engl. J. Med. 2019, 380, 2449. 31216400 10.1056/NEJMra1810762

[21] R. Basu , D. Pearson , B. Malig , R. Broadwin , R. Green , Epidemiology 2012, 23, 813. 23007039 10.1097/EDE.0b013e31826b7f97

[22] J. F. Bobb , Z. Obermeyer , Y. Wang , F. Dominici , JAMA, J. Am. Med. Assoc. 2014, 312, 2659. 10.1001/jama.2014.15715 PMC4319792 25536257

[23] C. J. Gronlund , A. Zanobetti , J. D. Schwartz , G. A. Wellenius , M. S. O'Neill , Environ. Health Perspect. 2014, 122, 1187. 24905551 10.1289/ehp.1206132 PMC4216145

[24] C. Sorensen , C. Howard , P. Prabhakaran , G. Horton , R. Basu , BMJ 2022, 378, 070762. 10.1136/bmj-2022-070762 35944909

[25] H. Khraishah , B. Alahmad , R. L. Ostergard , A. AlAshqar , M. Albaghdadi , N. Vellanki , M. M. Chowdhury , S. G. Al‐Kindi , A. Zanobetti , A. Gasparrini , S. Rajagopalan , Nat. Rev. Cardiol. 2022, 19, 798. 35672485 10.1038/s41569-022-00720-x

[26] S. Kehler , S. J. Birchall , Environment. Sci. Pol. 2023, 146, 144.

[27] O. Jay , A. Capon , P. Berry , C. Broderick , R. de Dear , G. Havenith , Y. Honda , R. S. Kovats , W. Ma , A. Malik , N. B. Morris , L. Nybo , S. I. Seneviratne , J. Vanos , K. L. Ebi , The Lancet 2021, 398, 709. 10.1016/S0140-6736(21)01209-5 34419206

[28] Y. Wu , S. Li , Q. Zhao , B. Wen , A. Gasparrini , S. Tong , A. Overcenco , A. Urban , A. Schneider , A. Entezari , A. Maria Vicedo‐Cabrera , A. Zanobetti , A. Analitis , A. Zeka , A. Tobias , B. Nunes , B. Alahmad , B. Armstrong , B. Forsberg , S. C. Pan , C. C. Íñiguez , C. Ameling , C. De la Cruz Valencia , C. Åström , D. Houthuijs , D. Van Dung , D. Royé , E. Indermitte , E. Lavigne , F. Mayvaneh , et al., The Lancet Planet Health 2022, 6, E410. 35550080 10.1016/S2542-5196(22)00073-0 PMC9177161

[29] A. Gasparrini , B. Armstrong , Epidemiology 2011, 22, 68. 21150355 10.1097/EDE.0b013e3181fdcd99 PMC3324776

[30] L. Madaniyazi , B. Armstrong , A. Tobias , M. N. Mistry , M. L. Bell , A. Urban , J. Kyselý , N. Ryti , I. Cvijanovic , C. F. S. Ng , D. Roye , A. M. Vicedo‐Cabrera , S. Tong , E. Lavigne , C. Íñiguez , S. N. P. da Silva , J. Madureira , J. J. K. Jaakkola , F. Sera , Y. Honda , A. Gasparrini , M. Hashizume , R. Abrutzky , F. Acquaotta , B. Alahmad , A. Analitis , H. K. Carlsen , G. Carrasco‐Escobar , M. de Sousa Zanotti Stagliorio Coelho , V. Colistro , et al., The Lancet Planetary Health 2024, 8, 86.

[31] K. L. Ebi , A. Capon , P. Berry , C. Broderick , R. de Dear , G. Havenith , Y. Honda , R. S. Kovats , W. Ma , A. Malik , N. B. Morris , L. Nybo , S. I. Seneviratne , J. Vanos , O. Jay , The Lancet 2021, 398, 698. 10.1016/S0140-6736(21)01208-3 34419205

[32] World Meteorological Organization , State of Climate Services‐Health, 2023. https://library.wmo.int/viewer/68500/download?file=1335_WMO‐Climate‐services‐Health_en.pdf&type=pdf&navigator=1 (accessed: September 2025).

[33] P. Masselot , M. Mistry , J. Vanoli , R. Schneider , T. Iungman , D. Garcia‐Leon , J. C. Ciscar , L. Feyen , H. Orru , A. Urban , S. Breitner , V. Huber , A. Schneider , E. Samoli , M. Stafoggia , F. de'Donato , S. Rao , B. Armstrong , M. Nieuwenhuijsen , K. Aunan , The Lancet Planetary Health 2023, 7, 271. 10.1016/S2542-5196(23)00023-2 36934727

[34] K. R. van Daalen , C. Tonne , J. C. Semenza , J. Rocklöv , A. Markandya , N. Dasandi , S. Jankin , H. Achebak , J. Ballester , H. Bechara , T. M. Beck , M. W. Callaghan , B. M. Carvalho , J. Chambers , M. C. Pradas , O. Courtenay , S. Dasgupta , M. J. Eckelman , Z. Farooq , P. Fransson , E. Gallo , O. Gasparyan , N. Gonzalez‐Reviriego , I. Hamilton , R. Hänninen , C. Hatfield , K. He , A. Kazmierczak , V. Kendrovski , H. Kennard , et al., Lancet Public Health 2024, 9, 495.

[35] S. Campbell , T. A. Remenyi , C. J. White , F. H. Johnston , Health and Place 2018, 53, 210. 30189362 10.1016/j.healthplace.2018.08.017

[36] World Health Organization , Report of a *Scoping Meeting* for the *Selection* of *Indicators* to *Monitor* the *Impact* of *Extreme Heat* on *Maternal*, *Newborn* and *Child H*ealth, World Health Organization, Geneva, Switzerland 2024.

[37] B. Armstrong , Epidemiology 2006, 17, 624. 17028505 10.1097/01.ede.0000239732.50999.8f

[38] A. Gasparrini , B. Armstrong , M. G. Kenward , Statist. Med. 2010, 29, 2224. 10.1002/sim.3940 PMC2998707 20812303

[39] A. Gasparrini , Stat. Med. 2014, 33, 881. 24027094 10.1002/sim.5963 PMC4098103

[40] S. Greenland , Int. J. Epidemiol 2004, 33, 1389. 15319402 10.1093/ije/dyh276

[41] A. Gasparrini , M. Leone , Med. Res. Methodol. 2014, 14, 55. 10.1186/1471-2288-14-55 PMC4021419 24758509

[42] J. Schwartz , Epidemiology 2001, 12, 55.11138820

[43] D. Shindell , Y. Zhang , M. Scott , M. Ru , K. Stark , K. L. Ebi , GeoHealth 2020, 4, 2019GH000234. 10.1029/2019GH000234 PMC7125937 32258942

[44] A. Gasparrini , B. Armstrong , Med. Res. Methodol. 2013, 13. 10.1186/1471-2288-13-1 PMC3599933 23297754

[45] D. Jackson , R. Riley , I. R. White , Stat. Med. 2011, 30, 2481. 21268052 10.1002/sim.4172 PMC3470931

[46] A. Gasparrini , B. Armstrong , M. G. Kenward , Stat. Med. 2012, 31, 3821. 22807043 10.1002/sim.5471 PMC3546395

[47] A. Gasparrini , Y. Guo , M. Hashizume , E. Lavigne , A. Zanobetti , J. Schwartz , A. Tobias , S. Tong , J. Rocklöv , B. Forsberg , M. Leone , M. De Sario , M. L. Bell , Y.‐L. L. Guo , C.‐F. Wu , H. Kan , S.‐M. Yi , M. de Sousa Zanotti Stagliorio Coelho , P. H. N. Saldiva , Y. Honda , H.o Kim , B. Armstrong , Lancet 2015, 386, 369. 26003380 10.1016/S0140-6736(14)62114-0 PMC4521077

[48] Y. Guo , A. Gasparrini , B. G. Armstrong , B. Tawatsupa , A. Tobias , E. Lavigne , M. D. Coelho , X. Pan , H.o Kim , M. Hashizume , Y. Honda , Y.‐L. L. Guo , C.‐F.u Wu , A. Zanobetti , J. D. Schwartz , M. L. Bell , M. Scortichini , P. Michelozzi , K. Punnasiri , S. Li , L. Tian , S. D. O. Garcia , X. Seposo , A. Overcenco , A. Zeka , P. Goodman , T. N. Dang , D. V. Dung , F. Mayvaneh , P. H. N. Saldiva , et al., Environ. Health Perspect. 2017, 125.

[49] È. Martínez‐Solanas , M. Quijal‐Zamorano , H. Achebak , D. Petrova , J. M. Robine , F. R. Herrmann , X. Rodó , J. Ballester , Lanc. Planet. Health 2021, 5, 446. 10.1016/S2542-5196(21)00150-9 34245715

[50] Y. Honda , M. Kondo , G. McGregor , H. Kim , Y. L. Guo , Y. Hijioka , M. Yoshikawa , K. Oka , S. Takano , S. Hales , R. S. Kovats , Environment. Health Prevent. Med. 2014, 19, 56. 10.1007/s12199-013-0354-6 PMC3890078 23928946

[51] S. M. Labib , Sci. Total Environ. 2024, 928, 172387. 38608883 10.1016/j.scitotenv.2024.172387

[52] J.‐M. Robine , S. L. K. Cheung , S. Le Roy , H. Van Oyen , C. Griffiths , J.‐P. Michel , F. R. Herrmann , Comptes. Rendus. Biol. 2008, 331, 171. 10.1016/j.crvi.2007.12.001 18241810

[53] A. Saucy , M. S. Ragettli , D. Vienneau , K. de Hoogh , L. Tangermann , B. Schäffer , J. M. Wunderli , N. Probst‐Hensch , M. Röösli , Sci. Total Environ. 2021, 790, 147958. 34098271 10.1016/j.scitotenv.2021.147958

[54] M. Pascal , S. Goria , V. Wagner , M. Sabastia , A. Guillet , E. Cordeau , C. Mauclair , S. Host , Environment. Int. 2021, 151, 106441. 10.1016/j.envint.2021.106441 33640693

[55] H. Achebak , D. Devolder , J. Ballester , PLoS Med. 2018, 15, 1002617. 10.1371/journal.pmed.1002617 PMC6057624 30040838

[56] J. Y. Son , J. C. Liu , M. L. Bell , Environment. Res. Lett. 2019, 14, 073004, 10.1088/1748-9326/ab1cdb PMC12362558 40837670

[57] A. Bunker , J. Wildenhain , A. Vandenbergh , N. Henschke , J. Rocklöv , S. Hajat , R. Sauerborn , EBioMedicine 2016, 6, 258. 27211569 10.1016/j.ebiom.2016.02.034 PMC4856745

[58] J. Yang , P. Yin , J. Sun , B. Wang , M. Zhou , M. Li , S. Tong , B. Meng , Y. Guo , Q. Liu , Sci. Total Environ. 2019, 649, 695. 30176480 10.1016/j.scitotenv.2018.08.332

[59] E. de Schrijver , M. Bundo , M. S. Ragettli , F. Sera , A. Gasparrini , O. H. Franco , A. M. Vicedo‐Cabrera , Environ. Health Perspect. 2022, 130, 037001. 35262415 10.1289/EHP9835 PMC8906252

[60] S. B. Henderson , K. E. McLean , M. J. Lee , T. Kosatsky , Environmental Epidemiology 2022, 6, 189. 10.1097/EE9.0000000000000189 PMC8835552 35169667

[61] H. Achebak , D. Devolder , J. Ballester , The Lancet Planetary Health 2019, 3, 297. 10.1016/S2542-5196(19)30090-7 31230996

[62] A. Gasparrini , P. Masselot , M. Scortichini , R. Schneider , M. N. Mistry , F. Sera , H. L. Macintyre , R. Phalkey , A. Maria Vicedo‐Cabrera , Articl. Lancet Planet Health 2022, 6, 557. 10.1016/S2542-5196(22)00138-3 35809585

[63] T. Leichtle , M. Kühnl , A. Droin , C. Beck , M. Hiete , H. Taubenböck , Urban Climate 2023, 49, 101522.

[64] Q. Zhao , S. Li , T. Ye , Y. Wu , A. Gasparrini , S. Tong , A. Urban , A. M. Vicedo‐Cabrera , A. Tobias , B. Armstrong , D. Royé , E. Lavigne , F. de'Donato , F. Sera , H. Kan , J. Schwartz , M. Pascal , N. Ryti , P. Goodman , Y. Guo , PLoS Med. 2024, 21, 1004364. 10.1371/journal.pmed.1004364 PMC11093289 38743771

[65] S. E. Kim , M. Hashizume , B. Armstrong , A. Gasparrini , K. Oka , Y. Hijioka , A. M. Vicedo‐Cabrera , Y. Honda , Environment. Health Perspect. 2023, 131, 1004364. 10.1289/EHP11444 PMC10181675 37172196

[66] W. T. Huang , P. Masselot , E. Bou‐Zeid , S. Fatichi , A. Paschalis , T. Sun , A. Gasparrini , G. Manoli , Nat. Commun. 2023, 14, 7438. 37978178 10.1038/s41467-023-43135-z PMC10656443

[67] P. Murage , S. Hajat , R. S. Kovats , Environment. Epidemiol. 2017, 1, 005. 10.1097/EE9.0000000000000005 PMC7608908 33195962

[68] A. M. Vicedo‐Cabrera , B. Forsberg , A. Tobias , A. Zanobetti , J. Schwartz , B. Armstrong , A. Gasparrini , Am. J. Epidemiol. 2016, 183, 286. 26811244 10.1093/aje/kwv205 PMC4753281

[69] a) W. Lee , Y. Kim , F. Sera , A. Gasparrini , R. Park , H. M. Choi , H. Kim , Lanc. Planet. Health 2020, 4, 512.

[70] M. Rai , M. Stafoggia , F. de'Donato , M. Scortichini , S. Zafeiratou , L. Vazquez Fernandez , S. Zhang , K. Katsouyanni , E. Samoli , S. Rao , E. Lavigne , Y. Guo , H. Kan , S. Osorio , J. Kyselý , A. Urban , H. Orru , M. Maasikmets , J. J. Jaakkola , S. Breitner , Environment. Int. 2023, 174, 107825. 10.1016/j.envint.2023.107825 36934570

[71] M. Stafoggia , P. Michelozzi , A. Schneider , B. Armstrong , M. Scortichini , M. Rai , S. Achilleos , B. Alahmad , A. Analitis , C. Åström , M. L. Bell , N. Calleja , H. Krage Carlsen , G. Carrasco , J. Paul Cauchi , M. DSZS Coelho , P. M. Correa , M. H. Diaz , A. Entezari , F. K. de’ Donato , Environment Int. 2023, 181, 108258. 10.1016/j.envint.2023.108258 PMC10702017 37837748

[72] J. Ballester , J.‐M. Robine , F. R. Herrmann , X. Rodó , Nat. Commun. 2011, 2, 358. 21694706 10.1038/ncomms1360

[73] S. Stewart , A. K. Keates , A. Redfern , J. J. V. McMurray , Nat. Rev. Cardiol. 2017, 14, 654. 28518176 10.1038/nrcardio.2017.76

[74] H. Achebak , D. Devolder , V. Ingole , J. Ballester , Nat. Commun. 2020, 11, 2457. 32433517 10.1038/s41467-020-16273-x PMC7239891

[75] H. L. Macintyre , C. Heaviside , X. Cai , R. Phalkey , Environment Int. 2021, 154, 106530. 10.1016/j.envint.2021.106530 PMC8543073 33895439

[76] A. Gasparrini , Y. Guo , F. Sera , A. M. Vicedo‐Cabrera , V. Huber , S. Tong , M. de Sousa Zanotti Stagliorio Coelho , P. H. Nascimento Saldiva , E. Lavigne , P. Matus Correa , N. Valdes Ortega , H. Kan , S. Osorio , J. Kyselý , A. Urban , J. J. Jaakkola , N. R. I. Ryti , M. Pascal , P. G. Goodman , A. Zeka , P. Michelozzi , M. Scortichini , M. Hashizume , Y. Honda , M. Hurtado‐Diaz , J. Cesar Cruz , X. Seposo , H.o Kim , A. Tobias , C. Iñiguez , et al., Lancet Planet Health 2017, 1, 360. 10.1016/S2542-5196(17)30156-0 PMC5729020 29276803

[77] Y. Guo , A. Gasparrini , S. Li , F. Sera , A. M. Vicedo‐Cabrera , M. de Sousa Zanotti Stagliorio Coelho , P. H. Saldiva , E. Lavigne , B. Tawatsupa , K. Punnasiri , A. Overcenco , P. M. Correa , N. V. Ortega , H. Kan , S. Osorio , J. J. Jaakkola , N. R. I. Ryti , P. G. Goodman , A. Zeka , P. Michelozzi , M. Scortichini , M. Hashizume , Y. Honda , X. Seposo , H.o Kim , A. Tobias , C. Íñiguez , B. Forsberg , D. O. Åström , Y. L. Guo , et al., PLoS Med. 2018, 15, 1002629. 10.1371/journal.pmed.1002629 PMC6067704 30063714

[78] V. Huber , L. Krummenauer , C. Peña‐Ortiz , S. Lange , A. Gasparrini , A. M. Vicedo‐Cabrera , R. Garcia‐Herrera , K. Frieler , Environ. Res. 2020, 186, 109447. 32302868 10.1016/j.envres.2020.109447

[79] D. García‐León , P. Masselot , M. N. Mistry , A. Gasparrini , C. Motta , L. Feyen , J.‐C. Ciscar , Lancet Public Health 2024, 9, 644,. 10.1016/S2468-2667(24)00179-8 39181156

[80] Q. Zhao , Y. Guo , T. Ye , S. Li , Q. Zhao , Y. Guo , T. Ye , A. Gasparrini , S. Tong , A. Overcenco , A. Urban , A. Schneider , A. Entezari , A. Maria Vicedo‐Cabrera , A. Zanobetti , A. Analitis , A. Zeka , A. Tobias , B. Nunes , S. Li , Lancet Planet Health 2021, 5, 415.

[81] World Health Organization , Heat and Health in the WHO European Region: Updated Evidence for Effective Prevention, World Health Organization, Geneva, Switzerland 2021.

[82] M. T. Moghadamnia , A. Ardalan , A. Mesdaghinia , A. Keshtkar , K. Naddafi , M. S. Yekaninejad , PeerJ 2017, 5, 3574. 10.7717/peerj.3574 PMC5546177 28791197

[83] K. Chen , S. Breitner , K. Wolf , R. Hampel , C. Meisinger , M. Heier , W. von Scheidt , B. Kuch , A. Peters , A. Schneider , A. Peters , H. Schulz , L. Schwettmann , R. Leidl , M. Heier , K. Strauch , Eur. Heart J. 2019, 40, 1600. 30859207 10.1093/eurheartj/ehz116

[84] Q. Yin , J. Wang , Z. Ren , J. Li , Y. Guo , Nat. Commun 2019, 10, 4640. 31604931 10.1038/s41467-019-12663-y PMC6789034

[85] A. Tobías , M. Hashizume , Y. Honda , F. Sera , C. F. Ng , Y. Kim , D. Roye , Y. Chung , T. N. Dang , H.o Kim , W. Lee , C. Íñiguez , A. Vicedo‐Cabrera , R. Abrutzky , Y. Guo , S. Tong , M. D. Coelho , P. H. Saldiva , E. Lavigne , P. M. Correa , N. V. Ortega , H. Kan , S. Osorio , J. Kyselý , A. Urban , H. Orru , E. Indermitte , J. J. Jaakkola , N. R. I. Ryti , M. Pascal , et al., Environ Epidemiol 2021, 5, 169.

[86] M. N. Mistry , R. Schneider , P. Masselot , D. Royé , B. Armstrong , J. Kyselý , H. Orru , F. Sera , S. Tong , É. Lavigne , A. Urban , J. Madureira , D. García‐León , D. Ibarreta , J. C. Ciscar , L. Feyen , E. de Schrijver , M. de Sousa Zanotti Stagliorio Coelho , M. Pascal , A. Gasparrini , Sci. Rep. 2022, 12, 5178. 35338191 10.1038/s41598-022-09049-4 PMC8956721

[87] J. Ballester , K. R. van Daalen , Z. Y. Chen , H. Achebak , J. M. Antó , X. Basagaña , R. Lowe , Lancet Region. Health–Europe 2024, 36, 100779. 10.1016/j.lanepe.2023.100779 PMC10769891 38188278

[88] T. Iungman , S. Khomenko , E. P. Barboza , M. Cirach , K. Gonçalves , P. Petrone , T. Erbertseder , H. Taubenböck , T. Chakraborty , M. Nieuwenhuijsen , The Lancet Planetary Health 2024, 8, 489. 10.1016/S2542-5196(24)00120-7 38969476

[89] R. C. Estoque , M. Ooba , X. T. Seposo , T. Togawa , Y. Hijioka , K. Takahashi , S. Nakamura , Nat. Commun. 2020, 11, 1581. 32221303 10.1038/s41467-020-15218-8 PMC7101384

[90] J. C. Semenza , Lancet Region. Health 2021, 9, 100231. 10.1016/j.lanepe.2021.100231 PMC8495299 34642677

[91] F. Marando , M. P. Heris , G. Zulian , A. Udías , L. Mentaschi , N. Chrysoulakis , D. Parastatidis , J. Maes , Sustainable Cities and Society 2022, 77, 103564.

[92] S. M. Labib , S. Lindley , J. J. Huck , Sci. Total Environ. 2021, 789, 147919. 34062470 10.1016/j.scitotenv.2021.147919

[93] N. A. Errett , C. Hartwell , J. M. Randazza , A. Nori‐Sarma , K. R. Weinberger , K. R. Spangler , Y. Sun , Q. H. Adams , G. A. Wellenius , J. J. Hess , BMC Public Health 2023, 23, 811. 37138325 10.1186/s12889-023-15757-x PMC10154751

[94] J. Vanoli , M. N. Mistry , L. C. de , A. Libardi , P. Masselot , R. Schneider , C. F. S. Ng , L. Madaniyazi , A. Gasparrini , J. Expos. Sci. Environment. Epidemiol. 2024, 34, 1012. 10.1038/s41370-023-00635-w PMC11618064 38191925

[95] J. Nawaro , L. Gianquintieri , A. Pagliosa , G. M. Sechi , E. G. Caiani , Public Health Reviews 2023, 44, 1606266. 37908198 10.3389/phrs.2023.1606266 PMC10613660

[96] P. J. Robinson , J. Appl. Meteorol. Climatol. 2001, 40, 762.

[97] *World Health Organization* Climate Change and Workplace Heat Stress: Technical Report and Guidance, World Health Organization, Geneva, Switzerland 2025. 10.1177/10482911251390889 PMC12804399 41442273

[98] T. Clima , W. Te , Guidelines on the Definition and Characterization of Extreme Weather and Climate Events, World Meteorological Organization, Geneva, Switzerland 2023.

[99] I. D. Stewart , T. R. Oke , Bull. Americ. Meteorolog. Soc. 2012, 93, 1879.

[100] J. Nawaro , L. Gianquintieri , A. Pagliosa , G. M. Sechi , E. G. Caiani , Populat. Environment 2024, 46, 25.

[101] C. Heaviside , S. Vardoulakis , X. M. Cai , UK. Environment. Health: Global Access Sci. Source 2016, 15, 49.

[102] A. Hsu , G. Sheriff , T. Chakraborty , D. Manya , Nat. Commun. 2021, 12, 1. 34035248 10.1038/s41467-021-22799-5 PMC8149665

[103] M. H. Browning , A. Rigolon , Int. J. Environ. Res. Public Health 2018, 15, 1541. 30037037 10.3390/ijerph15071541 PMC6068800

[104] H. Ohanyan , L. Portengen , A. Huss , E. Traini , J. W. Beulens , G. Hoek , J. Lakerveld , R. Vermeulen , Environ. Int. 2022, 158, 107015. 34991269 10.1016/j.envint.2021.107015

[105] A. Altmann , L. Tolosi , O. Sander , T. Lengauer , Bioinformatics 2010, 26, 1340. 20385727 10.1093/bioinformatics/btq134

[106] B. M. Greenwell , 2017, R J9, 421.

[107] S. M. Lundberg , S. I. Lee , (Eds.: I. Guyon , U. Von Luxburg , S. Bengio , H. Wallach , R. Fergus , S. Vishwanathan , R. Garnett ), Advances in Neural Information Processing Systems,Curran Associates, Inc., 2017, 30, https://proceedings.neurips.cc/paper_files/paper/2017/file/8a20a8621978632d76c43dfd28b67767‐Paper.pdf.

[108] L. Gianquintieri , D. Oxoli , E. G. Caiani , M. A. Brovelli , Chemosphere 2024, 352, 141438. 38367880 10.1016/j.chemosphere.2024.141438

[109] A. K. Leist , M. Klee , J. H. Kim , D. H. Rehkopf , S. P. Bordas , G. Muniz‐Terrera , S. Wade , Sci. Adv. 2022, 8, abk1942. 10.1126/sciadv.abk1942 PMC9581488 36260666

[110] J. Petch , S. Di , W. Nelson , Can. J. Cardiol. 2022, 38, 204. 34534619 10.1016/j.cjca.2021.09.004

[111] T. L. Wiemken , R. R. Kelley , Annu. Rev. Public Health 2020, 41, 21. 31577910 10.1146/annurev-publhealth-040119-094437

[112] Y. Nohara , K. Matsumoto , H. Soejima , N. Nakashima , Comput. Methods Prog. Biomed. 2022, 214, 106584. 10.1016/j.cmpb.2021.106584 34942412

